# The Hemogenic Competence of Endothelial Progenitors Is Restricted by *Runx1* Silencing during Embryonic Development

**DOI:** 10.1016/j.celrep.2016.05.001

**Published:** 2016-05-26

**Authors:** Alexia Eliades, Sarah Wareing, Elli Marinopoulou, Muhammad Z.H. Fadlullah, Rahima Patel, Joanna B. Grabarek, Berenika Plusa, Georges Lacaud, Valerie Kouskoff

**Affiliations:** 1 Cancer Research UK Stem Cell Hematopoiesis Group, Cancer Research UK Manchester Institute, The University of Manchester, Manchester M20 4BX, UK; 2 Cancer Research UK Stem Cell Biology Group, Cancer Research UK Manchester Institute, The University of Manchester, Manchester M20 4BX, UK; 3 Faculty of Life Sciences, The University of Manchester, Manchester M13 9PT, UK

## Abstract

It is now well-established that hematopoietic stem cells (HSCs) and progenitor cells originate from a specialized subset of endothelium, termed hemogenic endothelium (HE), via an endothelial-to-hematopoietic transition. However, the molecular mechanisms determining which endothelial progenitors possess this hemogenic potential are currently unknown. Here, we investigated the changes in hemogenic potential in endothelial progenitors at the early stages of embryonic development. Using an ETV2::GFP reporter mouse to isolate emerging endothelial progenitors, we observed a dramatic decrease in hemogenic potential between embryonic day (E)7.5 and E8.5. At the molecular level, *Runx1* is expressed at much lower levels in E8.5 intra-embryonic progenitors, while *Bmi1* expression is increased. Remarkably, the ectopic expression of *Runx1* in these progenitors fully restores their hemogenic potential, as does the suppression of BMI1 function. Altogether, our data demonstrate that hemogenic competency in recently specified endothelial progenitors is restrained through the active silencing of *Runx1* expression.

## Highlights

• ETV2^+^ progenitors from E8.5 embryos have very low hemogenic potential
• E8.5 ETV2^+^ progenitors specifically lack *Runx1* expression
• Ectopic expression of *Runx1* confers hemogenic potential to E8.5 ETV2^+^ progenitors
• *Runx1* silencing in ETV2^+^ endothelial progenitors is mediated by BMI1

## Graphical Abstract

**Figure:**
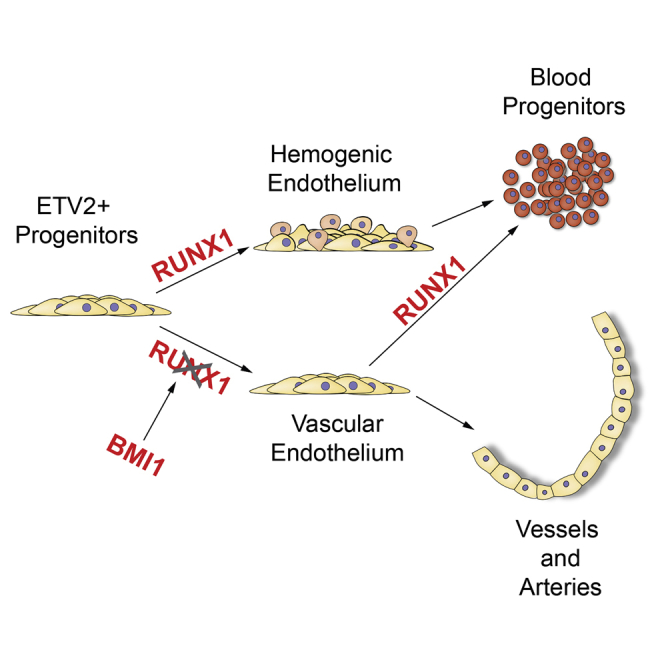

## Teaser

Hematopoiesis originates from specialized HE at specific stages of embryonic development. Eliades et al. demonstrate that RUNX1 repression is critical for suppressing hemogenic competency in endothelial progenitors at the E8.5 developmental stage and that this is achieved through the silencing of *Runx1* transcription by a BMI1-mediated mechanism.

## Introduction

Hematopoiesis emerges early in the vertebrate embryo and occurs in three major distinct waves (Costa et al., 2012). The two first waves take place in the extra-embryonic yolk sac (YS) between embryonic day (E)7.0 and E9.0 and give rise to primitive erythrocytes, macrophages, and megakaryocytes (Palis et al., 1999). The E7.5 primitive wave is rapidly followed by a definitive wave that begins at E8.25 with the generation of erythro-myeloid progenitors in the YS (Frame et al., 2015, McGrath et al., 2015, Palis et al., 1999). The first site of intra-embryonic hematopoiesis is the E9.5 para-aortic splanchnopleura (P-Sp), which further develops into the aorta-gonad-mesonephros (AGM) region. The AGM is the site where the first hematopoietic stem cells (HSCs) with long-term reconstituting and multi-lineage capacity emerge by E10.5 (Medvinsky et al., 1993, Müller et al., 1994). Fully functional HSCs are also detected a day later in the fetal liver (FL), placenta, and YS (Gekas et al., 2005, Medvinsky and Dzierzak, 1996).

It has long been proposed that blood and endothelial lineages originate from a common mesoderm progenitor. Early studies pointed to the close proximity of blood and endothelial cells in the blood island of the YS (Sabin, 1920). More recently, studies have demonstrated the existence of a common progenitor termed “hemangioblast” emerging from the primitive streak and expressing the T-box transcription factor Brachyury (*T*) and the fetal liver kinase receptor (*Flk1*) (Huber et al., 2004). Cell-fate tracing studies have established the endothelial origin of hematopoietic cells from a transient cell population termed “hemogenic endothelium” (HE) (Boisset et al., 2010, Jaffredo et al., 1998, Zovein et al., 2008). Additionally, using an in vitro system recapitulating YS hematopoiesis, it was shown that the hemangioblast also gave rise to hematopoietic progenitors via an HE intermediate (Lancrin et al., 2009). This HE cell population is characterized by the expression of FLK1, the endothelial tyrosine kinase receptor TIE2 (TEK), c-KIT, and VE-cadherin. Initially HE cells are flat, forming adherent tight endothelial cores, but as they undergo endothelial-to-hematopoietic transition (EHT), they acquire a round shape characteristic of circulating blood cells (Lancrin et al., 2009). We recently showed that *Etv2*, a member of the ETS (E26 transformation-specific) family of transcription factors, marks a subset of mesoderm FLK1^+^ cells as well as the next stage of blood specification, the HE. Additionally using a knockout approach, our study revealed the essential role of *Etv2* for the generation of HE (Wareing et al., 2012a). We and others also demonstrated the importance of this ETS factor during mesoderm specification, using a conditional deletion approach (Kataoka et al., 2011, Wareing et al., 2012b). ETV2 expression is required in FLK1^+^ cells for the initiation of the hematopoietic program via the activation of the key downstream target *Scl*, which, in turn, is critical for the development of HE (Lancrin et al., 2009, Wareing et al., 2012b). Furthermore, ETV2 deficiency in the mouse embryos resulted in a complete absence of all blood progenitors and vascular network (Lee et al., 2008, Sumanas et al., 2008, Wareing et al., 2012b). The transcription factor *Runx1* was also shown to be critical during hematopoietic specification. Although the HE emerges independently of *Runx1*, this factor is crucial for the subsequent formation of hematopoietic progenitors (Chen et al., 2009, Lancrin et al., 2009). Moreover, single-cell data generated from *Runx1 +23* enhancer-GFP reporter mice revealed activation of the hematopoietic program early in GFP^+^ cells, with a concomitant loss of the endothelial program (Swiers et al., 2013).

The characterization of mesoderm-derived cells at the onset of hematopoiesis allows the identification of the molecular mechanisms that control cell-fate choice toward hemogenic competence in endothelial cells. We and others have demonstrated that ETV2 expression marks FLK1^+^ cells, giving rise to all endothelial and hematopoietic derivatives (Kataoka et al., 2011, Wareing et al., 2012a). To date, it is still not understood how some endothelial progenitors are endowed with hemogenic competency while others are not. It remains to be demonstrated whether specification toward endothelial or hematopoietic fate is already pre-determined in specific subsets of ETV2^+^FLK1^+^ mesoderm or whether one program is dominant over the other, hence representing a default fate that needs to be suppressed. While the hemogenic potential of ETV2^+^FLK1^+^ cells has been shown at E7.5, it is not known whether ETV2^+^FLK1^+^ cells at later stages of development harbor the same characteristics. In this study, we sought to compare the hemogenic potential of ETV2^+^FLK1^+^ cells isolated from E7.5 and E8.5 embryos, using an ETV2::GFP reporter mouse (Wareing et al., 2012a). We observed that E8.5 ETV2::GFP^+^FLK1^+^CD41^−^ cells had minimal hemogenic potential compared to their E7.5 counterpart. Microarray and single-cell gene expression analysis suggested that this differential hemogenic potential might be due to a lack of *Runx1* expression. Remarkably, the ectopic expression of *Runx1* was able to redirect E8.5 ETV2::GFP^+^FLK1^+^ endothelial progenitors toward a hematopoietic fate. These data not only highlight the plasticity of ETV2^+^FLK1^+^ progenitors in developing embryos but also suggest that hematopoiesis is a fate that is actively suppressed by the silencing of *Runx1* to allow endothelial specification.

## Results

### ETV2 Expression Marks Similar Immuno-phenotypic Populations with Distinct Hematopoietic Potential

**Figure 1:**
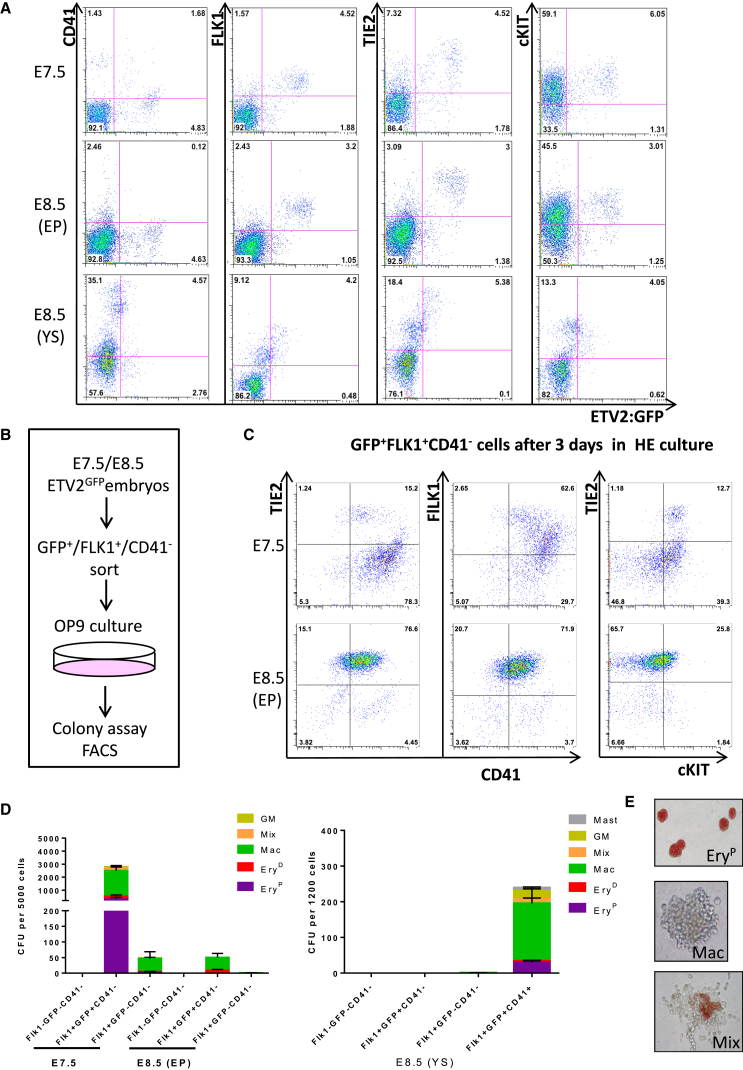
ETV2::GFP-Expressing FLK1 Cells Have Different Hematopoietic Potentials at Different Developmental Stages (A) FACS analysis for the expression of endothelial and hematopoietic markers at the E7.5 and E8.5 developmental stages. EP: embryo proper; YS: yolk sac. (B) Schematic representation of the experimental design. (C) FACS analysis of the sorted GFP^+^FLK1^+^CD41^−^ cells after three days in HE culture conditions. (D) Clonogenic assay for hematopoietic progenitors of FLK1^−^GFP^−^CD41^−^, FLK1^+^GFP^+^CD41^−^, FLK1^+^GFP^−^CD41^−^, and FLK1^+^GFP^+^CD41^+^ populations after three days in culture. Error bars represent 1 SEM from three independent experiments. CFU, colony-forming units; GM, granulocyte-macrophage. (E) Representative images of hematopoietic colonies from semisolid clonogenic assays. Ery^P^, primitive erythrocytes; Ery^D^, definitive erythrocytes; Mac, macrophages, Mix, granulocyte-erythroid-monocyte-macrophage (GEMM)/granulocyte-erythroid-macrophage (GEM). See also Figure S1.

We previously generated an ETV2::GFP transgenic mouse line in which GFP marks *Etv2*-expressing cells; using this mouse model, we showed that, at E7.5, GFP expression marked the YS wave of HE (Wareing et al., 2012a). A large fraction of ETV2::GFP^+^ cells in E7.5 embryos co-expressed c-KIT, TIE2, and FLK1, all marking the HE subset. Interestingly, ETV2::GFP^+^ cells from the embryo proper (EP) at E8.5 also co-expressed these markers, suggesting that this previously uncharacterized ETV2^+^ population could share similar hemogenic potential as the earlier E7.5 ETV2::GFP^+^ population (Figure 1A). ETV2::GFP^+^ cells within the YS of E8.5 embryos also co-expressed c-KIT, TIE2, and FLK1 but had much lower levels of GFP^+^ expression, suggesting a progressive downregulation of *Etv2* (Figure 1A). To compare the hemogenic potential of E7.5 and E8.5 (EP) ETV2::GFP^+^ cell populations, E7.5 and E8.5 embryos were harvested and cells were sorted based on their FLK1^+^GFP^+^CD41^−^ immuno-phenotype, followed by plating on OP9 stroma under conditions that support HE growth and maturation (Figure 1B; Figure S1A). After 3 days in culture, E7.5 cells upregulated CD41, indicative of hematopoietic emergence; the majority of the cultured cells also lost TIE2 expression and either maintained low levels or lost FLK1 expression. In contrast, while E8.5 cells also upregulated CD41 expression, they maintained high expression of the endothelial markers TIE2 and FLK1 (Figure 1C; Figure S1B). Only a small fraction of CD41^+^ cells lost expression of both endothelial markers at the E8.5 stage. This revealed that the majority of CD41^+^ cells derived from E8.5 ETV2::GFP^+^FLK1^+^ cells maintained an endothelial identity that might prevent further differentiation toward hematopoiesis. To explore the hematopoietic potential of these populations, cells were further replated in semisolid clonogenic assays. In line with the flow cytometry data, the E7.5 ETV2::GFP^+^FLK1^+^CD41^−^ population was able to generate hematopoietic progenitors at much higher levels when compared to its E8.5 (EP) and E8.5 (YS) counterparts (Figures 1D and 1E). These data show that the E8.5 HE does not reside within the E8.5 (YS) ETV2::GFP^+^ population but raise the possibility that the YS E8.5 HE could be derived from the E7.5 ETV2::GFP^+^ progenitors that displayed hemogenic potential. Finally, although E7.5 and E8.5 (EP) ETV2::GFP^+^FLK1^+^CD41^−^ populations both express FLK1, TIE2, and cKIT, markers also expressed in HE, they differ significantly in terms of their hemogenic potential.

**Figure 2:**
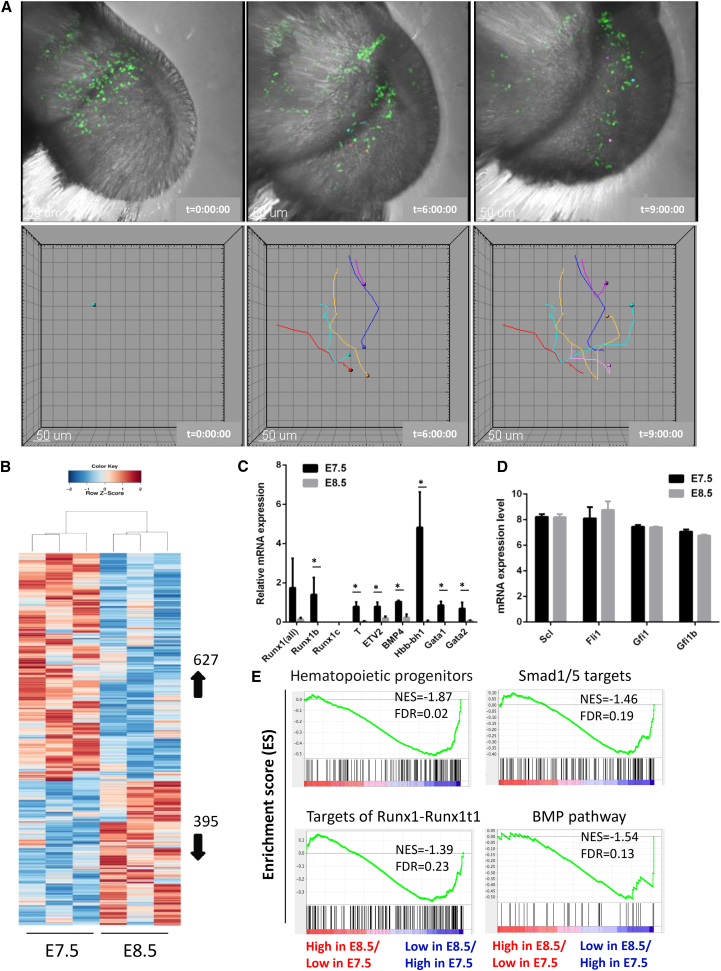
Live Imaging and Microarray Analysis Reveal Migratory Pattern and Differential Gene Expression of ETV2::GFP^+^ Cells at Different Developmental Stages (A) Still images from Movie S1 displaying the migratory path of some representative ETV2::GFP^+^ cells during the ex vivo culture of E7.5 embryos. Top: transmitted and GFP images of Z sections were stacked together to create a 3D reconstruction of the embryo. Bottom: tracking of ETV2::GFP^+^ cells through time. Color spots depict examples of some of the migrating ETV2::GFP^+^ cells. Scale bars, 50 μm, and time (t) is given in hr:min:sec. (B) Hierarchical clustering and heatmap for depicted genes in the GFP^+^FLK1^+^CD41^−^ cell population sorted from E7.5 and E8.5 (EP) ETV2::GFP embryos. (C) RT-qPCR for some of the differentially expressed genes found in the exon array. Three primer sets are used for detection of *Runx1* isoforms: *Runx1b*, *Runx1c*, and *Runx1b+c* (*Runx1all*). Data are means of triplicate experiments ± SD; two-tailed Student’s t test. ^∗^p < 0.05. (D) Exon array gene expression for hematopoietic transcription factors. Error bars indicate SD. (E) GSEA analysis depicting significantly enriched molecular processes in the gene set correlated with low gene expression in E8.5 compared to an E7.5 GFP^+^FLK1^+^CD41^−^ population. NES: normalized enrichment score; FDR: false discovery rate. See also Figure S2 and Movies S1, S2, S3, and S4.

The origin of the E8.5 ETV2::GFP^+^FLK1^+^ population still remains unclear. Previous work has shown that RUNX1^+^GATA1^−^ cells from E7.5 extra-embryonic tissue are able to migrate to the EP in a circulation-independent manner (Tanaka et al., 2014). This suggests that E7.5 extra-embryonic ETV2^+^ cells might similarly migrate to the embryo, giving rise to an intra-embryonic population. To address this issue, we performed time-lapse imaging of E7.5 *Etv2::gfp* embryos. In Figure 2A and Movies S1, S2, S3, and S4, representative examples of migrating ETV2::GFP^+^ cells are traced over time during the course of ex vivo embryonic development. These data revealed extra-embryonic ETV2^+^ cells at the boundary between extra- and intra-embryonic regions migrating to the intra-embryonic region as the embryo progresses toward the E8.5 stage. These findings demonstrate that E7.5 extra-embryonic ETV2^+^ cells are contributing to the pool of E8.5 intra-embryonic ETV2^+^ cells.

### E7.5 and E8.5 ETV2^+^FLK1^+^ Cells Show Differential Level of *Runx1* Expression

In order to define the underlying molecular mechanisms that contribute to the differential hemogenic potential, we performed a comparative global gene expression profiling of the E7.5 and E8.5 (EP) ETV2::GFP^+^FLK1^+^CD41^−^ populations. Principal-component analysis (PCA) confirmed close association among biological replicates but also clear differences between the two populations (Figure S2A). In addition, the gene expression profile of the ETV2^+^ progenitors was clearly separate from the Runx1^+^VECad^−^CD41^+^ primitive hematopoietic population, which represents a committed hematopoietic population (Tanaka et al., 2012b) (Figures S2A and S2B). Greater than 2-fold expression differences were observed in a total of 1,022 genes, of which 627 were expressed at higher levels and 395 showed lower levels in the E7.5, as compared to the E8.5 (EP) population (Figure 2B). Despite these differences in gene expression, both populations had similar expression of endothelial genes, indicating their shared endothelial identity (Figure S2C). Brachyury (*T*), an early mesoderm marker, and *Etv2* were expressed at lower levels in the E8.5 (EP) population (Figure 2C). In line with the direct relationship between these two populations, this might reflect the progressive downregulation of a hemangioblast mesoderm identity, as the ETV2^+^ cells further differentiate from their mesodermal origin and migrate from the extra-embryonic region toward the intra-embryonic region. Similarly indicative of a progressive transition, *Hbb-bh1*, a marker of primitive erythropoiesis taking place in the YS, was also expressed at lower levels in the E8.5 (EP) population. *Runx1* followed a similar pattern of expression and was, in fact, one of the few hematopoietic transcription factors differentially expressed between the two ETV2::GFP^+^FLK1^+^ populations, along with *Gata1* and *Gata2* (Figure 2C). This pattern of *Runx1* expression was linked to the activity of the proximal P2 promoter that generates the *Runx1b* isoform transcript, since the *Runx1c* isoform transcript (regulated by the distal P1 promoter) was absent in both populations (Figure 2B). *Runx1b* is the *Runx1* isoform that is specifically expressed in HE at the onset of EHT (Sroczynska et al., 2009). The transcription factors *Scl* and *Fli1*, which are part of a recursive triad along with *Gata2* (Pimanda et al., 2007b) known to control hematopoietic development, were expressed at similar levels in the two populations (Figure 2D). Gene set enrichment analysis (GSEA) revealed high enrichment for hematopoietic progenitors as well as SMAD1/5, BMP4, and Runx1-Runx1t1 pathways within genes that were expressed at higher levels in E7.5 cells, as compared to E8.5(EP) cells (Figure 2E). These results suggest that the BMP4-SMAD1/5 pathway and *Runx1* activation in E7.5 ETV2::GFP^+^FLK1^+^ cells could be implicated in the underlying mechanisms of the high hemogenic potential of this population at E7.5. Altogether, these data suggest a pre-eminent role for RUNX1 in the temporal control of the hemogenic ability of ETV2^+^ progenitor cells during early embryonic development.

### Heterogeneity within E7.5-E8.5 ETV2::GFP^+^ Progenitors

**Figure 3:**
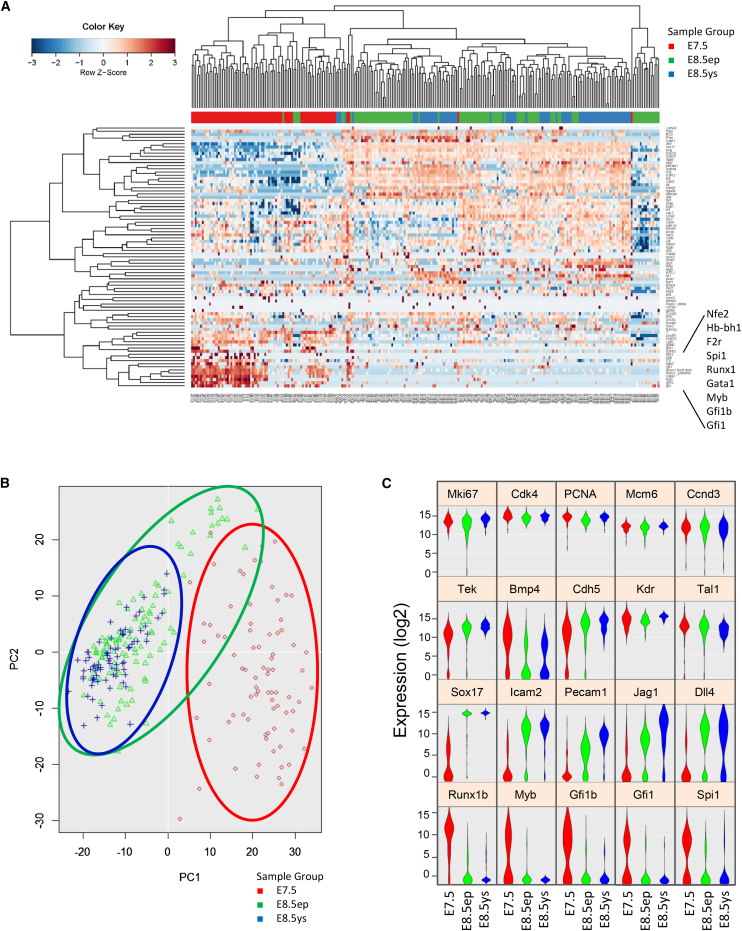
Single-Cell Gene Expression Analysis Reveals Heterogeneity in ETV2::GFP-Expressing FLK1 Cells (A) The Fluidigm platform was used for single-cell gene expression analysis as described in the Supplemental Experimental Procedures. Unsupervised hierarchical clustering heatmap for depicted genes. (B) PCA for the cells analyzed shows the degree of segregation among the three populations. (C) Frequency of distribution of the expression level for each gene shown as Violin plots. The width of the violin plot represents the frequency of cells at that expression level. See also Figure S3.

Given that FLK1 and ETV2 are also expressed in hemangioblast prior to HE emergence (Wareing et al., 2012a), the ETV2::GFP^+^FLK1^+^CD41^−^ sorted cells are likely to be heterogeneous in nature. In order to assess their degree of heterogeneity, we performed single-cell gene expression analysis on a total of 262 ETV2::GFP^+^FLK1^+^CD41^−^ single cells from E7.5, E8.5 (EP), and E8.5 (YS) ETV2::GFP^+^ embryos using a nanofluidic platform. A total of 96 genes were analyzed, including housekeeping genes, cell-cycle genes, and transcription factors involved in mesoderm, endothelial, and hematopoietic development. Unsupervised hierarchical clustering and PCA analysis showed that the E8.5 (EP) and E8.5 (YS) populations cluster closely together and are distinct from the E7.5 cells (Figures 3A and 3B). Heatmap and violin plots revealed a high and overall unimodal expression of cell-cycle genes *Mki67*, *Cdk4*, *Mcm6*, *Ccnd3*, and *Pcna*, suggesting that the higher hematopoietic potential of the E7.5 ETV2^+^FLK1^+^ cells was not due to increased proliferation. As expected, *Flk1* (*Kdr*) expression was also high in all populations, while *Bmp4* was downregulated in E8.5, a finding consistent with the global expression data. Similarly, while *Runx1b* was expressed at high levels in most E7.5 single cells (72 out of 80), only 24 out of the 103 E8.5 (EP) and 20 out of the 79 E8.5 (YS) single cells expressed this transcription factor (Figure 3C; Figure S3). Heterogeneity in the expression of hematopoietic genes such as *Ikzf2* and *Itga2b* was also observed in the E8.5 (EP) population, while E7.5 displayed an overall high expression pattern for a number of hematopoietic transcription factors. In contrast, the majority of the E8.5 cells analyzed had low or undetectable expression levels of hematopoietic genes such as *Myb* or *Spi1* (Figure 3C). Unsupervised hierarchical clustering for all genes showed that some E7.5 single cells clustered closer to the E8.5 (EP) cells rather than to the E7.5 population (Figure 3A). This E7.5 subset that clustered with the E8.5 population expressed *Runx1* (13 out of 17 cells), the majority of which (11 out of 13 Runx1^+^ cells) were *Gata1*^*−*^ and could represent the migrating RUNX1^+^GATA1^−^ cell population previously described (Tanaka et al., 2014). Interestingly, a small subset of cells in each population was negative for *Etv2* (Figure S3). The E7.5 *Etv2*^*−*^ cells expressed hematopoietic genes and could represent cells that progressed further toward hematopoiesis; the *Etv2*^*−*^ E8.5 (EP) and E8.5 (YS) cells did not express hematopoietic genes. A subset of *Etv2*^*−*^ cells, including E8.5 (EP) and E8.5 (YS) cells, expressed a strong endothelial signature and could represent cells further committed to the endothelial lineage. A third subset of *Etv2*^*−*^ cells only detected in E8.5 (EP) had low or undetectable expression of either endothelial or hematopoietic genes. These cells, only found in the EP, could represent progenitors differentiating toward cardiac fate, in line with a previous report demonstrating the necessity for *Etv2* silencing for cardiac differentiation (Schupp et al., 2014) (Figure S3). Altogether, these single-cell analyses not only confirmed data from the microarray analysis on the bulk population but also revealed the heterogeneity within ETV2::GFP^+^FLK1^+^CD41^−^ populations at different developmental stages.

### RUNX1 and ETV2 Are Co-expressed in E7.5 but Not in E8.5 Embryos

**Figure 4:**
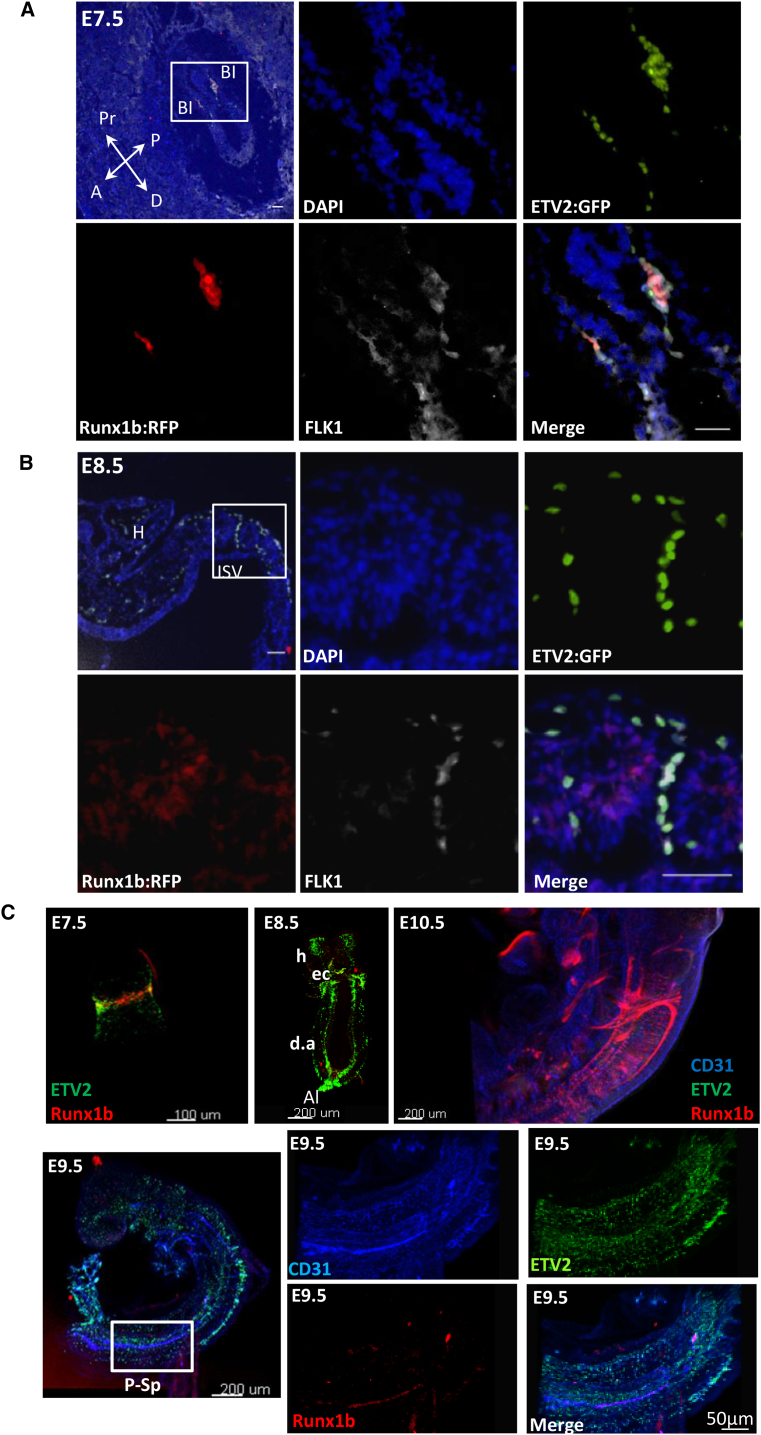
Expression Pattern of RUNX1b and ETV2 in Developing Embryos (A and B) Transversal section of *Etv2::gfp Runx1b::rfp* E7.5 (A) and E8.5 (B) embryos stained for ETV2::GFP (green), Runx1b::RFP (red), and FLK1 (white). Scale bars, 50 μm. (C) Whole-mount imaging for ETV2::GFP RUNX1b::RFP and CD31 for E7.5-E10.5 developmental stages. Pr: Proximal; D: Distal; A: Anterior; P: Posterior; BI: blood island; H: head; ISV: intersomitic vessel; da: dorsal aorta; ec: endocardium; and P-Sp: para-aortic splanchnopleura region. See also Figure S4 and Movies S5 and S6.

The global expression profiling and single-cell data analysis revealed a distinct pattern of *Runx1* mRNA expression between E7.5 and E8.5 ETV2::GFP^+^ cells. To further explore their expression pattern, we crossed *Etv2::gfp* (Wareing et al., 2012a) and *Runx1b::rfp* (Sroczynska et al., 2009) transgenic mice to concurrently assess the expression of both genes in situ. At E7.5, both RUNX1b::RFP and ETV2::GFP were found co-expressed in FLK1^+^ cells in the blood islands in the YS (Figures 4A and 4C; Movie S5). ETV2::GFP also marked cells migrating from the primitive streak toward the extra-embryonic mesoderm (Figure 4A). At E8.5, ETV2::GFP was no longer detected in the YS blood islands, whereas RUNX1b::RFP was expressed in FLK1^−^ cells, likely representing primitive blood cells (Figure S4A). The allantois was the only extra-embryonic tissue that retained high expression of ETV2::GFP but lacked expression of RUNX1b::RFP at E8.5 (Figure S4B). In the E8.5 (EP), ETV2::GFP had a broad pattern of expression, as it was found expressed along the dorsal aorta, the sprouting inter-somitic vessels, the cephalic mesenchyme, and the endocardium. ETV2::GFP^+^FLK1^+^ cells exhibited no or minimal expression of RUNX1b::RFP (Figures 4B and 4C; Movie S6). At E9.5, ETV2::GFP expression was downregulated but still widely expressed, while RUNX1b::RFP expression was observed within the P-Sp region. By E10.5, embryos no longer expressed ETV2::GFP, while RUNX1b::RFP was expressed in circulating blood cells (Figure 4C).

**Figure 5:**
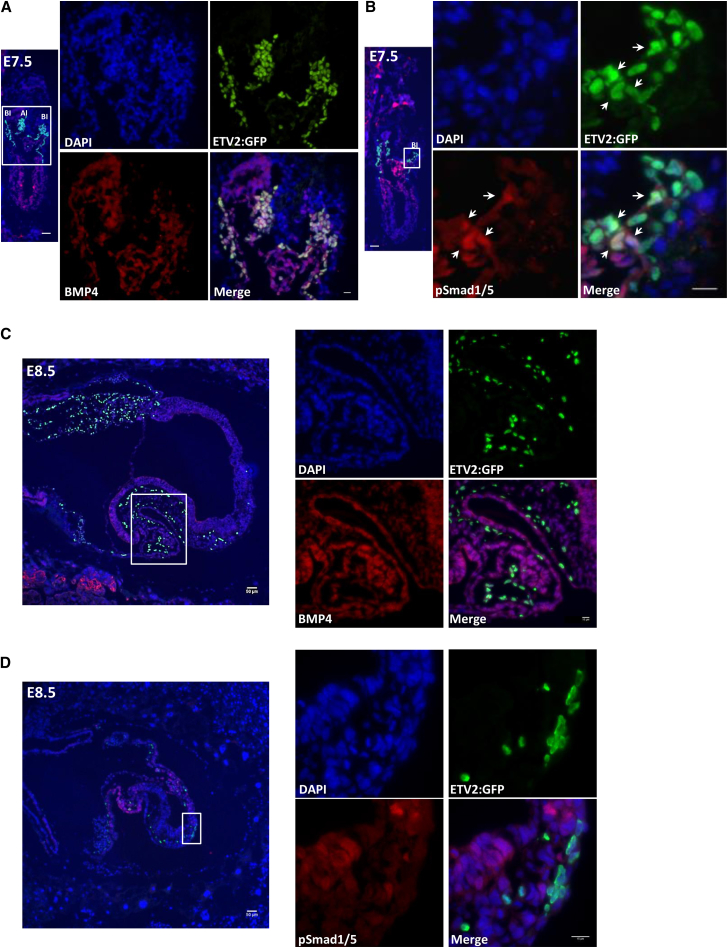
BMP4-pSMAD1/5 Activity in E7.5 Underlies the High Hematopoietic Potential of the ETV2::GFP Cells (A) E7.5 ETV2::GFP embryos were stained for GFP (green) and BMP4 (red). BI: blood island; Al: allantois. (B) Serial section of the same E7.5 embryo used in (A) was used for staining with GFP and pSMAD1/5. (C) E8.5 ETV2::GFP embryos were stained for GFP (green) and BMP4 (red). (D) E8.5 ETV2::GFP embryo stained for pSMAD1/5 (red). Scale bars, 50 μm for whole-embryo images and 15 μm for magnified images.

In the gastrulating embryo, Indian hedgehog is secreted from visceral endoderm and stimulates the expression of BMP4 from the extraembryonic mesoderm. BMP4, in turn, feeds back to mesodermal cells (in an autocrine or paracrine manner) to induce the hematopoietic and endothelial programs (Baron, 2001, Dyer et al., 2001). It was previously shown that BMP4 positively regulates the expression of *Runx1b* via the SMAD1/5 pathway (Pimanda et al., 2007a). Therefore, we hypothesized that ETV2::GFP^+^FLK1^+^ cells in the YS that expressed high levels of RUNX1b also expressed BMP4 and activated SMAD1/5. Indeed, at E7.5, BMP4 was detected at high levels in GFP^+^ cells in the blood islands and in the allantois (Figure 5A). Accordingly, part of the ETV2::GFP^+^ cells in the blood islands also showed staining for phosphorylated SMAD1/5 (Figure 5B). In contrast, E8.5 ETV2::GFP^+^ cells did not express detectable levels of either BMP4 or pSMAD1/5 (Figures 5C and 5D). Altogether, staining in E7.5-E8.5 embryos revealed a contrasting pattern of RUNX1 expression in ETV2^+^ cells. ETV2-expressing cells at E7.5 showed high expression of BMP4 and active SMAD1/5 that could underlie the mechanism of RUNX1b expression. In contrast, ETV2^+^ cells in the E8.5 (EP) did not express BMP4 or active Smad1/5 and lacked detectable expression of RUNX1b.

### *Runx1b* Ectopic Expression Restores the Hemogenic Potential of E8.5 ETV2::GFP^+^FLK1^+^ Cells

**Figure 6:**
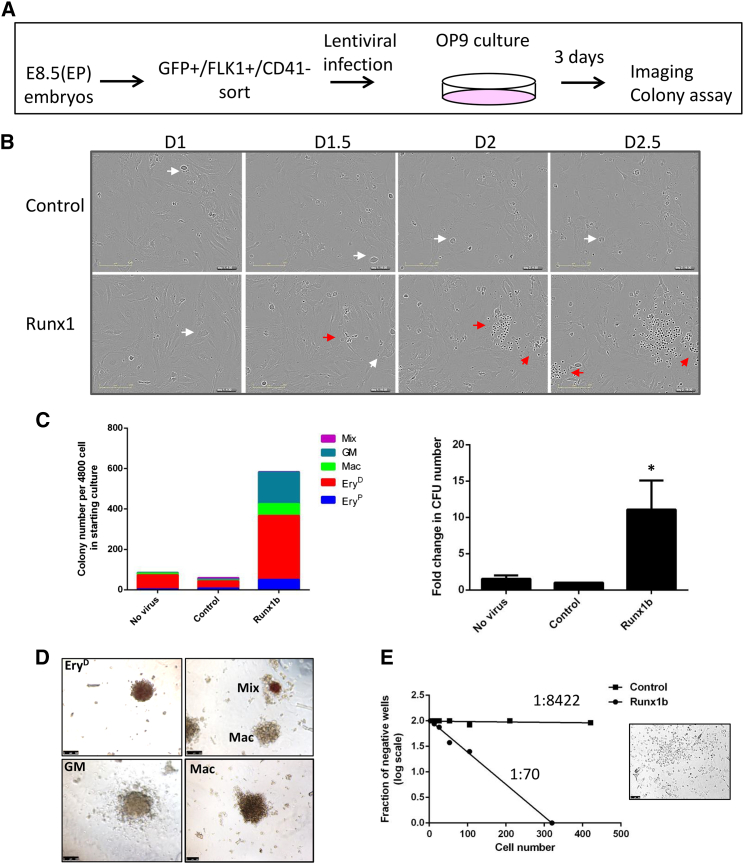
RUNX1b Ectopic Expression in E8.5 ETV2::GFP^+^FLK1^+^ Cells Enhances Their Hematopoietic Potential (A) Schematic representation of the experimental design. (B) Time-lapse imaging during the three-day OP9 culture of cells transduced with control or Runx1 lentivirus. White arrows indicate endothelial clusters; red arrows indicate hematopoietic cells. (C) Clonogenic assay for hematopoietic progenitors of control and Runx1b transduced cells after three days of culture on OP9 stroma layer. Left graph shows the number of hematopoietic colonies formed for each type of hematopoietic CFU. Right graph shows the overall change in CFU number per treatment. Error bars represent 1 SD from three independent experiments. ^∗^p < 0.05. Mix, GEMM/GEM; GM: granulocyte-macrophage; Mac: macrophages; Ery^P^: primitive erythrocytes; and Ery^D^: definitive erythrocytes. (D) Examples of hematopoietic colonies at day eight of replating in semisolid clonogenic assay. Scale bars represent 500 μm. (E) Left: limiting dilution assay (LDA) of hemogenic potential of FLK1^+^GFP^+^CD41^−^-sorted cells derived from E8.5 ETV2::GFP embryos and infected with either control or Runx1 lentivirus before plating on OP9 stoma. Right: example of hematopoietic cells emerging in OP9 cultures indicative of hemogenic endothelial potential in LDA. Data shown are representative of two independent experiments. See also Figure S5 and Movie S7.

Given that higher expression of *Runx1* in ETV2::GFP^+^FLK1^+^CD41^−^ cells correlated with higher hemogenic potential at E7.5 when compared to the E8.5 stage, we hypothesized that the expression of *Runx1b* might potentially restore the ability of E8.5 endothelial progenitor cells to produce hematopoietic cells. Therefore, we sorted E8.5 (EP) ETV2::GFP^+^FLK1^+^CD41^−^ cells, transduced them with *Runx1b* or control empty lentivirus, and tested their hemogenic potential (Figure 6A; Figures S5A and S5B). Live imaging revealed that, while both control and *Runx1*-treated cultures generated adherent endothelial cores, only *Runx1*-treated cells generated floating hematopoietic cells (Figure 6B; Movie S7). Interestingly, *Runx1*-treated cells gave rise to a 10-fold higher number of hematopoietic colonies compared to the control-treated cells (Figures 6C and 6D). Taken together, these data reveal that *Runx1* expression alone is sufficient to confer hematopoietic potential to ETV2^+^FLK1^+^ endothelial progenitors.

### *Runx1b* Increases the Frequency of E8.5 ETV2::GFP^+^FLK1^+^ Cells with HE Potential

The effect of *Runx1* expression on E8.5 ETV2::GFP^+^FLK1^+^CD41^−^ cells could be attributed either to an increase in the proliferation rate of the few HE cells present in this population or an increase in the number of cells that displayed HE potential. To address this, we performed western blot analysis on control and *Runx1*-treated E8.5 ETV2::GFP^+^FLK1^+^CD41^−^ cultures for proliferating cell nuclear antigen (PCNA), but no change in PCNA protein levels was detected (Figure S5B). Cell-cycle analysis also showed no substantial change in the percentage of cells in S phase (Figure S5C). These results suggest that RUNX1 is not mediating its effect through a change in proliferation. To determine the effect of *Runx1b* on the frequency of ETV2^+^FLK1^+^ cells that acquire HE potential, we performed a limiting dilution assay (LDA) on control and *Runx1*-treated cells. *Runx1*-transduced ETV2::GFP^+^FLK1^+^CD41^−^ cultures had a far greater frequency of cells giving rise to hematopoietic cells as compared to control (Figure 6E). In conclusion, the low frequency of hematopoietic cells generated by the control-transduced cells indicates that the HE potential of these cells is rare (1 in 8422). Once E8.5 ETV2^+^FLK1^+^ cells ectopically express *Runx1*, they show a dramatic increase in their capacity to give rise to hematopoietic colonies (1 in 70), an effect that is independent of the proliferative state of these cells. Altogether, these data suggest that the ability of ETV2::GFP^+^FLK1^+^CD41^−^ endothelium progenitors in the developing embryo to give rise to hematopoietic cells is mainly dependent on *Runx1* expression.

### BMI1 Inhibition De-represses *Runx1* Expression and Confers Hemogenic Potential to E8.5 ETV2::GFP Endothelial Progenitors

**Figure 7:**
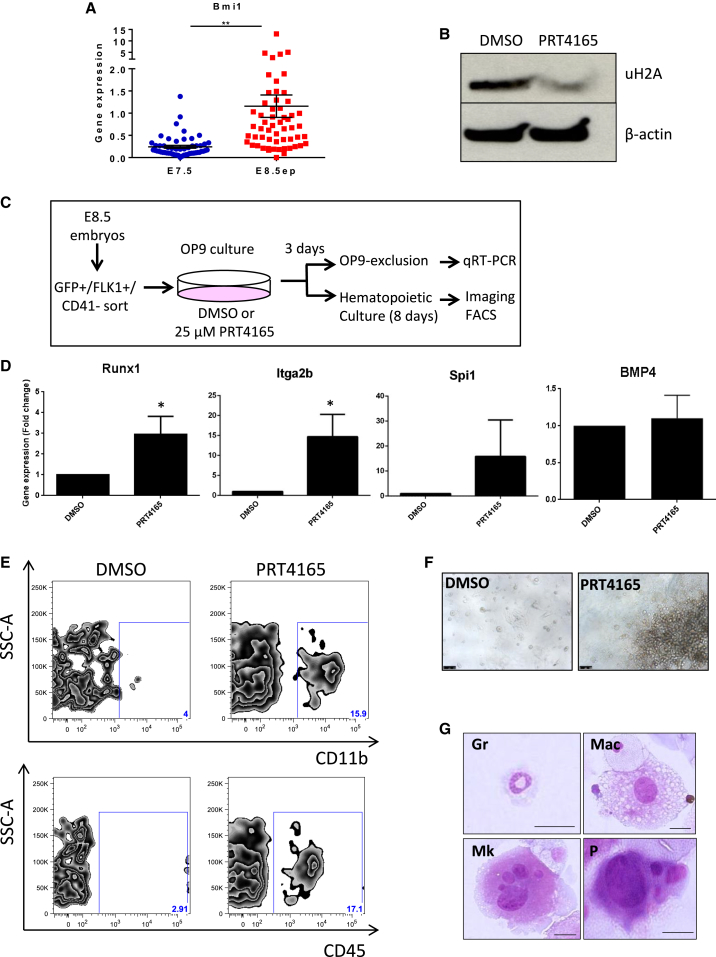
Inhibition of BMI1 De-represses *Runx1* Expression and Confers Hemogenic Potential to E8.5 ETV2::GFP Endothelial Progenitors (A) Single-cell gene expression analysis for *Bmi1* in E7.5 and E8.5 (EP) ETV2::GFP^+^FLK1^+^CD41^−^ progenitors. Error bars represent 1 SEM for at least 54 single cells. ^∗∗^p ≤ 0.001. (B) Western blot analysis for ubiquitinylated H2AK119, a downstream target of the PRC1 complex. FLK1^+^ sorted cells from day-3 embryoid body cultures were treated with 25 μM PRT4165 inhibitor or DMSO for 3 days prior to protein isolation. (C) Schematic representation of the experimental design. (D) RT-qPCR for E8.5 ETV2::GFP^+^FLK1^+^CD41^−^ cells cultured in HE conditions and treated with DMSO or 25 μM PRT4165 for 3 days prior to RNA extraction. Error bars represent 1 SEM for at least five experiments (expect for BMP4, n = 4). ^∗^p < 0.05. (E) FACS analysis of day-8 hematopoietic culture for the expression of hematopoietic markers. (F) Emergence of hematopoietic colonies after 8 days in hematopoietic culture conditions. Scale bars, 500 μm. (G) Representative May-Giemsa staining from PRT4165-treated cells after 8 days in hematopoietic culture conditions. Gr: granulocytes; Mac: macrophages; Mk: megakaryocyte; P: progenitor, and scale bars, 20 μm. See also Figure S6.

The single-cell gene expression analysis and ETV2::GFP embryo staining revealed that *Bmi1* was highly expressed in E8.5 intra-embryonic ETV2::GFP cells but was absent or expressed at low levels in the E7.5 ETV2::GFP population (Figure 7A; Figures S6A and S6B). BMI1 is a RING finger protein and an integral component of the Polycomb Repressive Complex 1 (PRC1) that mediates epigenetic silencing of targeted genes (Buchwald et al., 2006, Schwartz and Pirrotta, 2008). A previous study showed that *Runx1* was downregulated by BMI1 in K562 cells (Shen et al., 2014). Thus, we hypothesized that PRC1 represses *Runx1* transcription in E8.5 ETV2::GFP^+^ cells, thereby suppressing the hematopoietic transcriptional program and the HE potential of these progenitors. To test this, we first used PRT4165, a pharmacological inhibitor of the BMI1/RING1-mediated E3 ubiquitin ligase activity of PRC1 (Alchanati et al., 2009). PRT4165-mediated BMI1 inhibition was confirmed by a decrease in H2A^K119^ ubiquitination, a target of PRC1, in FLK1^+^ cells derived from day 3 embryoid body cultures (Figure 7B). To assess BMI1 inhibition on endothelial progenitors, E8.5 ETV2::GFP^+^FLK1^+^CD41^−^ cells were sorted and cultured on an OP9 stroma layer with PRT4165 or DMSO and tested for the expression of hematopoietic genes (Figure 7C). Interestingly, *Runx1* expression was upregulated upon BMI1 inhibition along with other hematopoietic genes, including *Itga2b* and *Spi1* (Figure 7D). Similarly, Bmi1 silencing, using small interfering RNA (siRNA), also resulted in an increase in the expression of hematopoietic genes (Figure S6C). BMP4, an upstream regulator of *Runx1* showed no significant change upon BMI1 inhibition and silencing. To determine the effect of BMI1 inhibition on their HE potential, PRT4165- or DMSO-treated cells were further cultured in a cocktail of cytokines to promote hematopoiesis. Fluorescence-activated cell sorting (FACS) analysis and May*-*Grünwald-Giemsa staining showed a strong increase in CD45^+^ and CD11b^+^ hematopoietic cells upon BMI1 inhibition (Figures 7E–7G). Altogether, these results indicate that PRC1 inhibition de-represses *Runx1* expression and, consequently, activates the hematopoietic program in E8.5 ETV2::GFP^+^ endothelial progenitors conferring them HE potential.

We also assessed the expression of BMI1 in the YS and AGM at E8.5 and E10.5, respectively (Figure S7). BMI1 was expressed in CD31^+^ cells and at moderate levels in CD41^+^ cells (Figures S7A and S7B). Given that there are no markers to distinguish HE from endothelial progenitors, it is not clear whether BMI1 expression is restricted to endothelial progenitors. Similarly to BMI1, BMP4 was also detected in both CD31^+^ and CD41^+^ E8.5, but its expression was reduced in ETV2:GFP^+^ progenitors (Figures S7C and S7D). In the E10.5 AGM, BMI1 was detected in both the CD31^+^cKIT^−^ endothelial layer and in CD31^−^cKIT^+^ emerging hematopoietic clusters (Figure S7E). This suggests that, by E10.5, other mechanisms beyond the BMI1-polycomb complex are involved in repressing the hematopoietic program in endothelial cells, as previously shown (Lizama et al., 2015). Overall, these data suggest that the BMI1-mediated mechanism of *Runx1* suppression is restricted to the E8.5 intra-embryonic ETV2::GFP^+^ progenitor population.

## Discussion

A large body of work has emerged recently regarding the role of ETV2 at the onset of blood and endothelium specification (Oh et al., 2015). The expression of ETV2 is restricted to a narrow window of embryonic development between E7.0 and E9.5 (Koyano-Nakagawa et al., 2012, Wareing et al., 2012a). In the absence of ETV2, mesodermal progenitors differentiate to the cardiomyocyte fate concomitantly with a complete defect in hematopoiesis and vasculogenesis (Liu et al., 2013, Palencia-Desai et al., 2011). It is now clear that the expression of ETV2 in FLK1^+^ is essential for the progression to hematopoiesis and endothelium (Wareing et al., 2012a). However, the molecular mechanisms that confer hemogenic competence to ETV2-expressing FLK1^+^ progenitors are poorly understood.

Here, we investigated the hemogenic potential of ETV2^+^FLK1^+^ progenitor populations at the early stages of embryogenesis, using a well-characterized ETV2::GFP reporter mouse (Wareing et al., 2012a). We found that both the E7.5 extra-embryonic and E8.5 intra-embryonic ETV2::GFP^+^ cells share similar expression of HE markers while lacking expression of CD41. However, when cultured under conditions that promote HE maintenance and growth, the E8.5 population appeared to maintain an endothelial identity, while the E7.5 population downregulated the expression of endothelial markers in CD41^+^ emerging cells. Accordingly, E8.5 cells gave rise to very few hematopoietic colonies in clonogenic assays, compared to the E7.5 population. Global gene expression analysis identified *Runx1* as one of the few hematopoietic transcription factors highly differentially expressed between the two ETV2::GFP^+^FLK1^+^CD41^−^ populations, a finding confirmed by in situ immunofluorescence and single-cell expression analysis. To examine the functional role of *Runx1* in ETV2::GFP^+^FLK1^+^CD41^−^ cells, we ectopically expressed *Runx1* in the population isolated from E8.5 embryos. Interestingly, *Runx1* expression dramatically increased the hemogenic potential of these cells, demonstrating that the expression of *Runx1* on its own is sufficient to confer hemogenic competence to ETV2^+^ progenitors. To investigate the relationship between the E7.5 and E8.5 ETV2^+^ populations, we performed time-lapse microscopy. Live-imaging analysis showed the migration of ETV2^+^ cells from the extra-embryonic site to the intra-embryonic site, suggesting that at least a subset of the E8.5 ETV2^+^ cells arise from E7.5 extra-embryonic progenitors. Our data, however, did not determine whether the ETV2-migrating cells previously expressed or not RUNX1. Whether *Runx1* is transiently expressed at an earlier stage of development contributing later to endothelium and hematopoiesis has been previously investigated. Tanaka et al. published a study showing the migration of RUNX1^+^GATA1^−^ cells from extra- to intra-embryonic endothelium (Tanaka et al., 2014). Further work also suggested the early and transient expression of RUNX1 in progenitors contributing to late hematopoiesis (Samokhvalov et al., 2007, Tanaka et al., 2012a). However, the conclusions of these studies remain disputed; therefore, further work will be required to fully address this issue.

Our study suggests that *Runx1* expression is repressed in endothelial progenitors at the early stages of embryonic development via a BMI1-dependent mechanism to limit hematopoietic potential to a subset of progenitors. Our results demonstrate that ectopic expression of *Runx1* alone is sufficient to confer hemogenic competence to E8.5 FLK1^+^ETV2^+^ endothelial progenitors. These findings reveal that the entire molecular framework required for blood specification is present in these endothelial progenitors, with the notable exception of RUNX1. A chromatin immunoprecipitation (ChIP)-sequencing study has shown the importance of RUNX1 in orchestrating the formation of hematopoietic-specific transcription binding pattern at the onset of hematopoiesis (Lichtinger et al., 2012). This study showed that *Runx1* induction in *Runx1*^*−/−*^ HE results in a rapid shift in the binding of SCL and FLI1 on hematopoietic genes, an increase in histone acetylation, and the formation of new transcription factor complexes. It is possible that, in E8.5 endothelial progenitors, these RUNX1-mediated changes do not occur due to a lack of *Runx1* expression, hence resulting in the maintenance of the endothelial program.

An earlier study focused on the mechanisms by which hematopoiesis is restricted to the HE domain during embryonic development; HOXA3 expression in the intra-embryonic vasculature was shown to restrain hematopoietic differentiation of endothelial progenitors by downregulating *Runx1* expression (Iacovino et al., 2011). Our work proposes an additional mechanism of hemogenic competence restriction. We show that BMI1, a subunit of the PRC1 complex, is highly expressed in E8.5 ETV2::GFP^+^FLK1^+^CD41^−^ but not detectable in E7.5 ETV2::GFP^+^FLK1^+^CD41^−^ cells. BMI1 inhibition in the E8.5 population resulted in not only an increase in *Runx1* expression along with other hematopoietic genes but also an increased HE potential. The role of BMI1 in suppressing HE potential was shown here, using an ex vivo experimental approach. Future in vivo studies will need to be performed to fully confirm the role of BMI1 in this mechanism. While the role of BMI1 at later stages of hematopoiesis is well-established (Ding et al., 2012), its role in early endothelial progenitors was not previously addressed. Our data indicate that the BMI1 mechanism of *Runx1* repression is present only in a narrow window of development when ETV2^+^ progenitors are present at the E8.5 stage. The role of BMI1 in ETV2^+^ endothelial progenitors is clearly distinct from its role in committed hematopoietic cells. E8.5 ETV2^+^ cells are endothelial progenitors, E8.5 CD41^+^ YS cells are mostly committed primitive erythroid cells, including primitive and definitive progenitors (Ferkowicz et al., 2003), and E10.5 cluster cells are mostly HSCs and pre-HSCs (Boisset et al., 2015, Rybtsov et al., 2011, Taoudi et al., 2008). The recruitment of BMI1 to specific DNA regions for silencing is cell context dependent and is unlikely to be identical in endothelium, primitive erythroid cells and embryonic pre-HSCs/HSCs. In support of this cell-context dependency within cell populations of the hematopoietic system, an additional role for BMI1 was recently described in erythroid cells where BMI1 is implicated in the regulation of ribosome biogenesis (Gao et al., 2015), clearly different from its role in adult HSCs, where BMI1 is implicated in the silencing the *p16Ink4a p19Arf* locus (Park et al., 2003).

Our data identify an additional role for BMI1 in maintaining an endothelial fate in E8.5 *Etv2*-expressing progenitors. This finding suggests that, in E8.5 ETV2^+^ progenitors, the hematopoietic program is silenced via a PRC1-mediated repression mechanism. The ectopic re-expression of *Runx1* or its de-repression via BMI1 inhibition or silencing results in the acquisition of hemogenic fate, suggesting that the hematopoietic program needs to be repressed for further progression of the endothelial fate during embryonic development. It remains to be determined whether BMI1 has a direct or indirect effect on *Runx1*. A previous study has shown that SUZ12, a component of the PRC2, directly binds *Runx1* as well as *Bmp4* gene loci (Lee et al., 2006). Given the synergistic action of PRC1 and PRC2, it is likely that the BMI1-containing PRC1 complex mediates the repression of *Runx1.* Our results show that *Bmp4* downregulation is independent of BMI1-mediated action, but it remains to be determined whether PCR2 alone is implicated in this process. This molecular mechanism of differential specification to blood and endothelium is consistent with the proposed evolutionary development of the circulatory and blood system. While blood cells are found in all multicellular organisms of the animal kingdom, the presence of endothelial cells is restricted to the vascular system of the vertebrate phyla (Muñoz-Chápuli et al., 2005). A prevalent hypothesis for the evolutionary origin of endothelium proposes that the early endothelium of vertebrates might have arisen from circulatory amoebocytes or hemal blood cells through the repression of the blood program and the acquisition of epithelial-type morphology (Muñoz-Chápuli et al., 2005, Pascual-Anaya et al., 2013). Our findings lend support to this hypothesis and suggest that this evolutionary mechanism might still be preserved in early ETV2^+^ endothelial progenitors.

Our study suggests that, during embryonic development, blood and endothelium cell fates are differentially established through the active repression of RUNX1 in common progenitors to allow endothelial fate to proceed. This mechanism of alternative repression of cell fates during early mesoderm specification is reminiscent of the repression of the cardiomyocyte fate by SCL in prospective HE (Van Handel et al., 2012). Altogether, our results raise questions about the plasticity of early mesoderm progenitors and the window of opportunity for differential cell fates that might help us devise more efficient protocols for the derivation of repopulating blood progenitors in vitro.

## Experimental Procedures

### Mice

Timed matings were set up between *Etv2::gfp* (Wareing et al., 2012a) males and ICR females or between *Etv2::gfp* and *Runx1b::rfp* mice as described in Sroczynska et al. (2009) study, except that the hCD4 reporter was replaced with red fluorescent protein [RFP] heterozygote mice. The morning of the vaginal plug was considered as E0.5. All animal work was performed according to the United Kingdom Animal Scientific Procedures Act 1896.

### Embryo Cell Culture

Sorted populations were seeded on irradiated OP9 stroma cells in Iscove’s modified Dulbecco’s medium (IMDM) containing 10% fetal bovine serum (FBS), 4 mM L-glutamine, 50 U/ml penicillin/streptomycin (Pen/Strep), 0.6% transferrin, 2% leukemia inhibitory factor (LIF), 48.75 μg/ml monothiolglycerol, 25 μg/ml ascorbic acid, 2% LIF, 1% kit ligand (KL) supernatant, 10 ng/ml oncostatin M, 1 ng/ml basic fibroblast growth factor (bFGF), and 1 μg/ml osteopontin. Where indicated, cells were cultured in semisolid clonogenic assay (Wareing et al., 2012a). BMI1/RING1A inhibitor PRT4165 (Tocris) was used at 25 μM.

### Lentiviral Transduction

PCR-amplified *Runx1b* and *Tomato* cDNAs separated by a 2A sequence (de Felipe et al., 1999) were cloned into a lentiviral vector containing the human EF1 promoter (Gilham et al., 2010). VSVg-pseudotyped lentiviral particles were produced and titered as previously described (Dull et al., 1998).

### Flow Cytometry

Single-cell suspensions were stained with FLK1-Biotin, CD41-APC, TIE2-PE, or cKIT-APCeF780 followed by Streptavidin-PECy7 (eBioscience). Cells were sorted using Aria II, Aria III, or Influx and analyzed with an LSRII cytometer (BD Biosciences).

### Statistical Analyses

Data were analyzed using a Student’s t test. Significant differences are ^∗^p < 0.05 and ^∗∗^p ≤ 0.001.

Additional information is available in the Supplemental Experimental Procedures.

## Author Contributions

A.E. designed and performed experiments, analyzed the data, and wrote the manuscript. S.W. and R.P. performed experiments; E.M. and M.Z.H.F. performed bioinformatics analysis, J.B.G. and B.P. assisted with microscopy; V.K. and G.L. designed and supervised the project, analyzed the data, and wrote the manuscript.

## Accession Numbers

The accession number for the microarray data reported in this study is GEO: GSE64377.

## Supplemental Information

Document S1. Supplemental Experimental Procedures and Figures S1–S7

Movie S1. Example 1, Live imaging of E7.5 *Etv2::gfp* embryo, Related to Figure S2

Movie S2. Example 2, Live imaging of E7.5 *Etv2::gfp* embryo, Related to Figure S2

Movie S3. Example 3, Live imaging of E7.5 *Etv2::gfp* embryo, Related to Figure S2

Movie S4. Example 4, Live imaging of E7.5 *Etv2::gfp* embryo, Related to Figure S2

Movie S5. 3D Reconstruction of z Stacks of E7.5 *Etv2::gfp Runx1b::rfp* Embryos Stained for CD31, in Magenta; ETV2::GFP, in Green; and RUNX1b::RFP, in Red, Related to Figure 4

Movie S6. 3D Reconstruction of z Stacks of E8.5 *Etv2::gfp Runx1b::rfp* Embryos Stained for ETV2::GFP, in Green, and RUNX1b::RFP, in Red, Related to Figure 4

Movie S7. Time-Lapse Imaging of E8.5 FLK1^+^GFP^+^CD41^−^ Sorted Cells on OP9 Monolayer Cultures Transduced with Control and Runx1 Lentivirus, Related to Figure 6

Document S2. Article plus Supplemental Information
