# Supplementary material for: The Hemogenic Competence of Endothelial Progenitors Is Restricted by Runx1 Silencing during Embryonic Development
Source: Cell Rep. 2016 May 26;15(10):2185–99. doi: 10.1016/j.celrep.2016.05.001 (PMC4906370; doi:10.1016/j.celrep.2016.05.001)
Supplement: Document S1. Supplemental Experimental Procedures and Figures S1–S7 [file mmc1.pdf]

Cell Reports, Volume *15*

Supplemental Information

The Hemogenic Competence of Endothelial Progenitors Is Restricted by *Runx1* Silencing during Embryonic Development

Alexia Eliades, Sarah Wareing, Elli Marinopoulou, Muhammad Z.H. Fadlullah, Rahima Patel, Joanna B. Grabarek, Berenika Plusa, Georges Lacaud, and Valerie Kouskoff

Supplemental Data

The hemogenic competence of endothelial progenitors is restricted by *Runx1* silencing during embryonic development

Alexia Eliades[1], Sarah Wareing[1], Elli Marinopoulou[2], Muhammad Z. H. Fadlullah[1], Rahima Patel[2], Joanna Grabarek[3], Berenika Plusa[3], Georges Lacaud[2*], Valerie Kouskoff[1*]

Author Information

[1] Cancer Research UK Stem Cell Hematopoiesis Group, Cancer Research UK Manchester Institute, The University of Manchester, M20 4BX, United Kingdom

[2] Cancer Research UK Stem Cell Biology Group, Cancer Research UK Manchester Institute, The University of Manchester, M20 4BX, United Kingdom

[3]Faculty of Life Sciences, The University of Manchester, M13 9PT, United Kingdom

Contact: Valerie Kouskoff, PhD. Cancer Research UK Stem Cell Hematopoiesis Group, Cancer Research UK Manchester Institute, The University of Manchester, M20 4BX, United Kingdom, email: Valerie.kouskoff@cruk.manchester.ac.uk

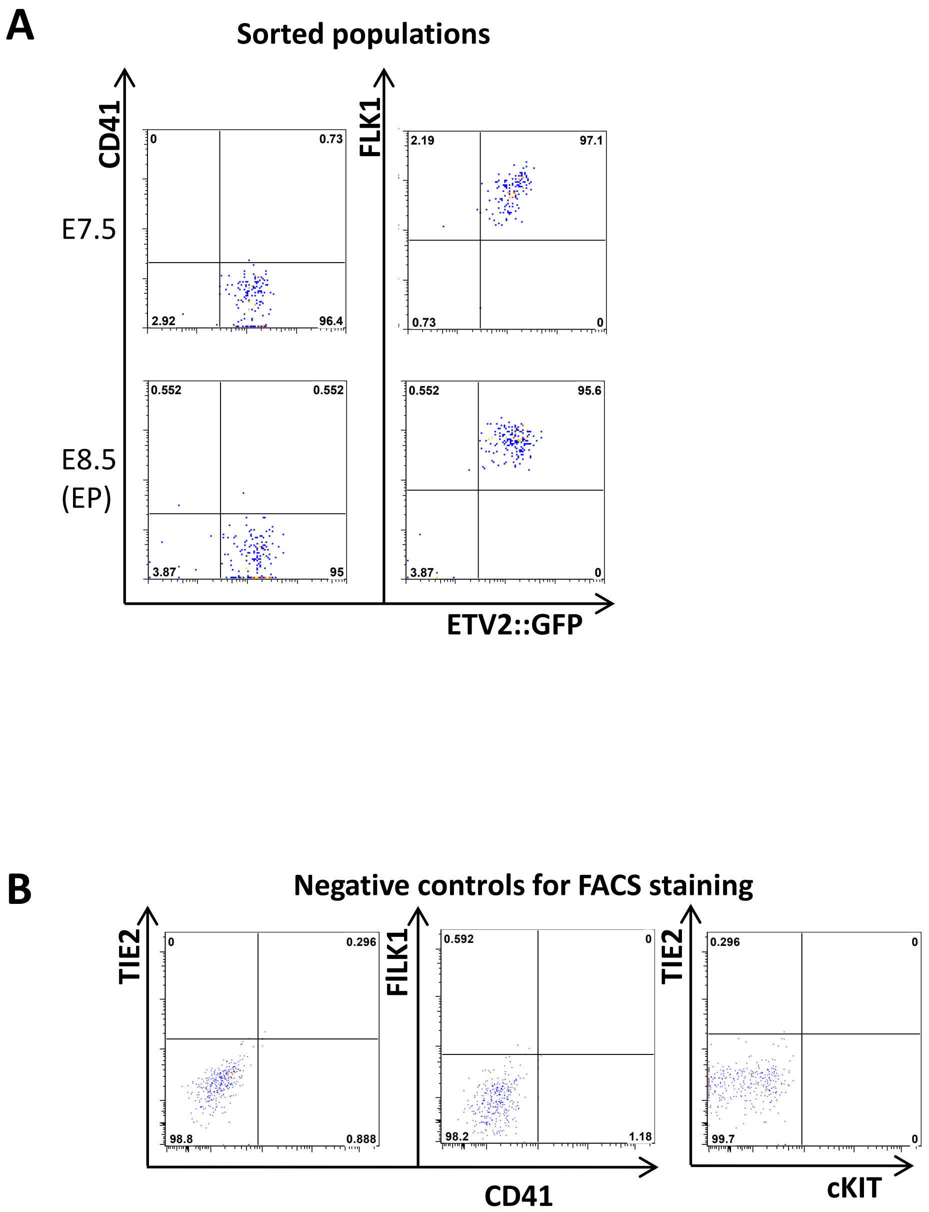

Figure S1. Immunophenitypic profile of ETV2:GFP+ embryo-derived cells. Related to Figure 1. **A.** FACS analysis of the sorted ETV2::GFP^{+}FLK1^{+}CD41^{-} cells before plating on OP9 stroma culture. **B.** Negative (non-stained) control related to Figure 1C.

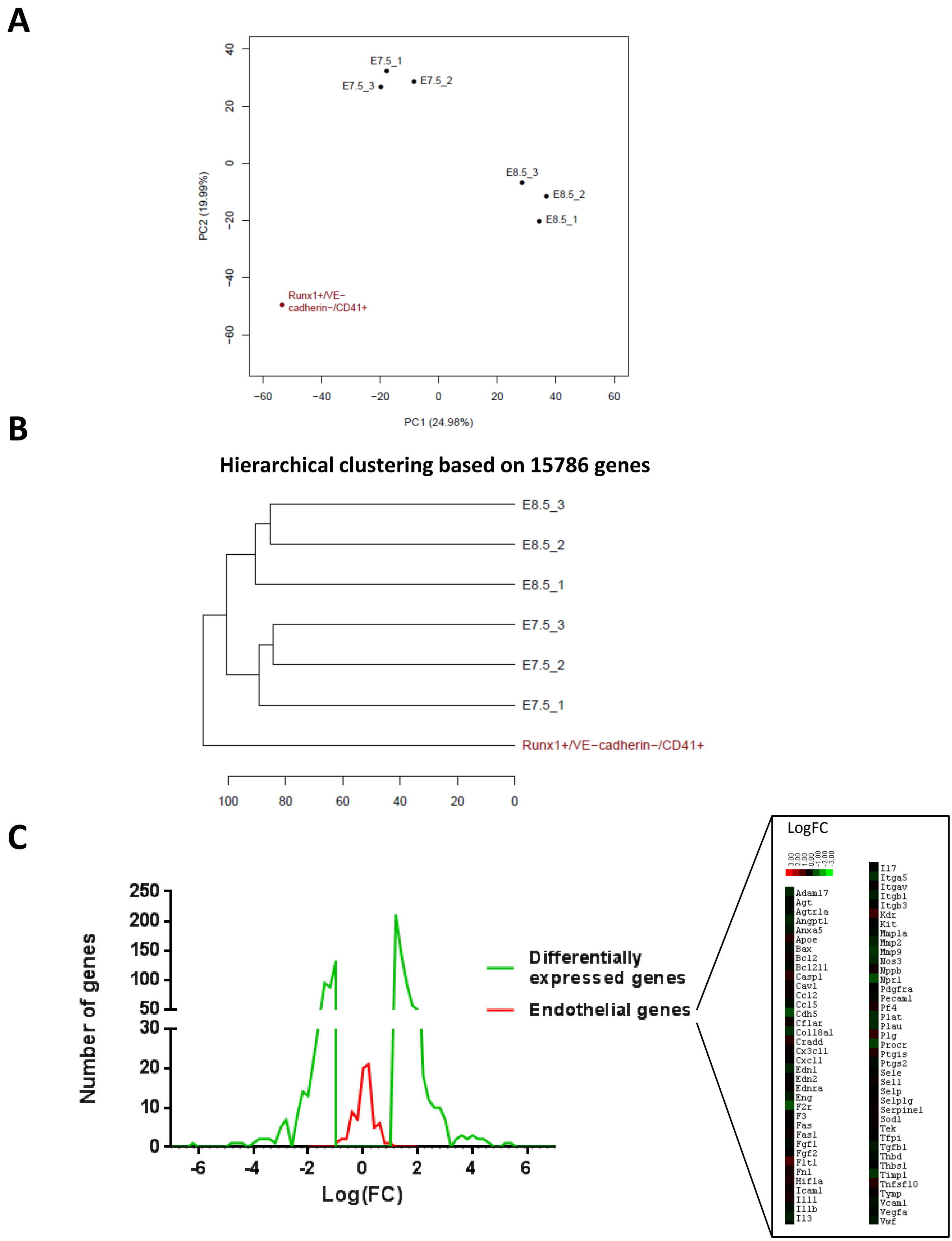

Figure S2. ETV2::GFP$^+$ transcriptome analysis at E7.5 and E8.5 developmental stage. Related to Figure 2. **A.** Principal Component Analysis (PCA) plot for the samples analyzed in the Affymetrix array (Figure 2) and sample Runx1$^+$VECad$^-$CD41$^+$ from Tanaka Y et al., 2012 (GSM721028). **B.** Hierarchical clustering based on 15786 genes expressed both in our Affymetrix array and GSM721028. **C.** Left: Histogram plot depicting the distribution of the fold-change in gene expression in logarithmic scale (logFC) for endothelial genes and differentially expressed genes. Right: Heatmap for the LogFC value for endothelial genes

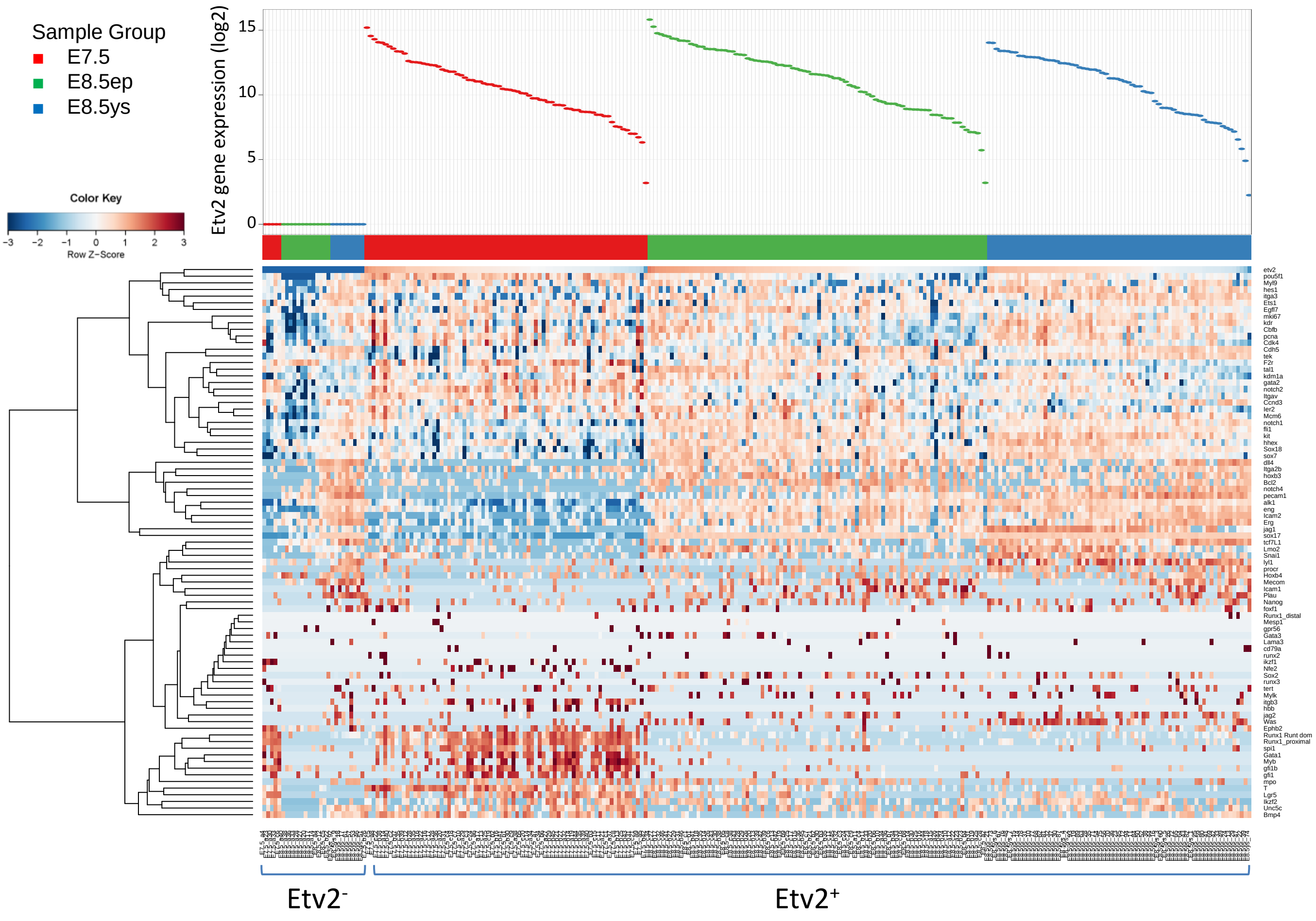

Figure S3. Single cell gene expression analysis of ETV2::GFP$^+$ cells. Related to Figure 3. Heat map for 83 genes analyzed in the ETV2::GFP$^+$FLK1$^+$CD41$^-$ cell populations sorted from E7.5 embryos, E8.5 embryo proper (ep) and E8.5 yolk sac (ys) *Etv2::gfp embryos.* Graph depicts *Etv2* gene expression (log2).

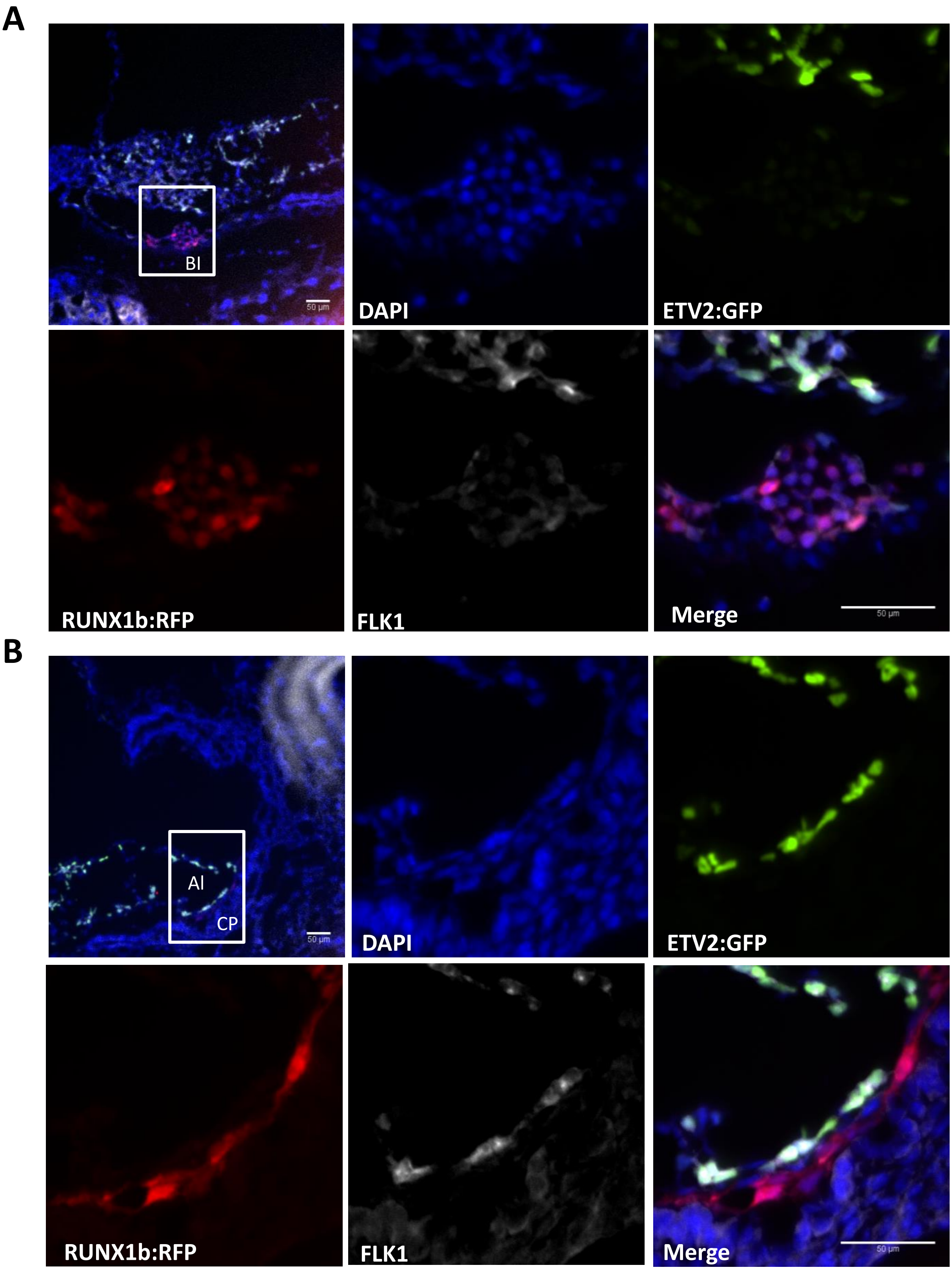

Figure S4. Expression of RUNX1b and ETV2 in extra-embryonic tissues at E8.5. Related to Figure 4. Transversal section of *Etv2::gfp Runx1b::rfp* E8.5 embryos stained for ETV2::GFP (green), RUNX1b::RFP (red) and FLK1 (white) **(A-B)**. BI=blood island, AL= Allantois, CP=Chorionic Plate. Scale bar=50µm.

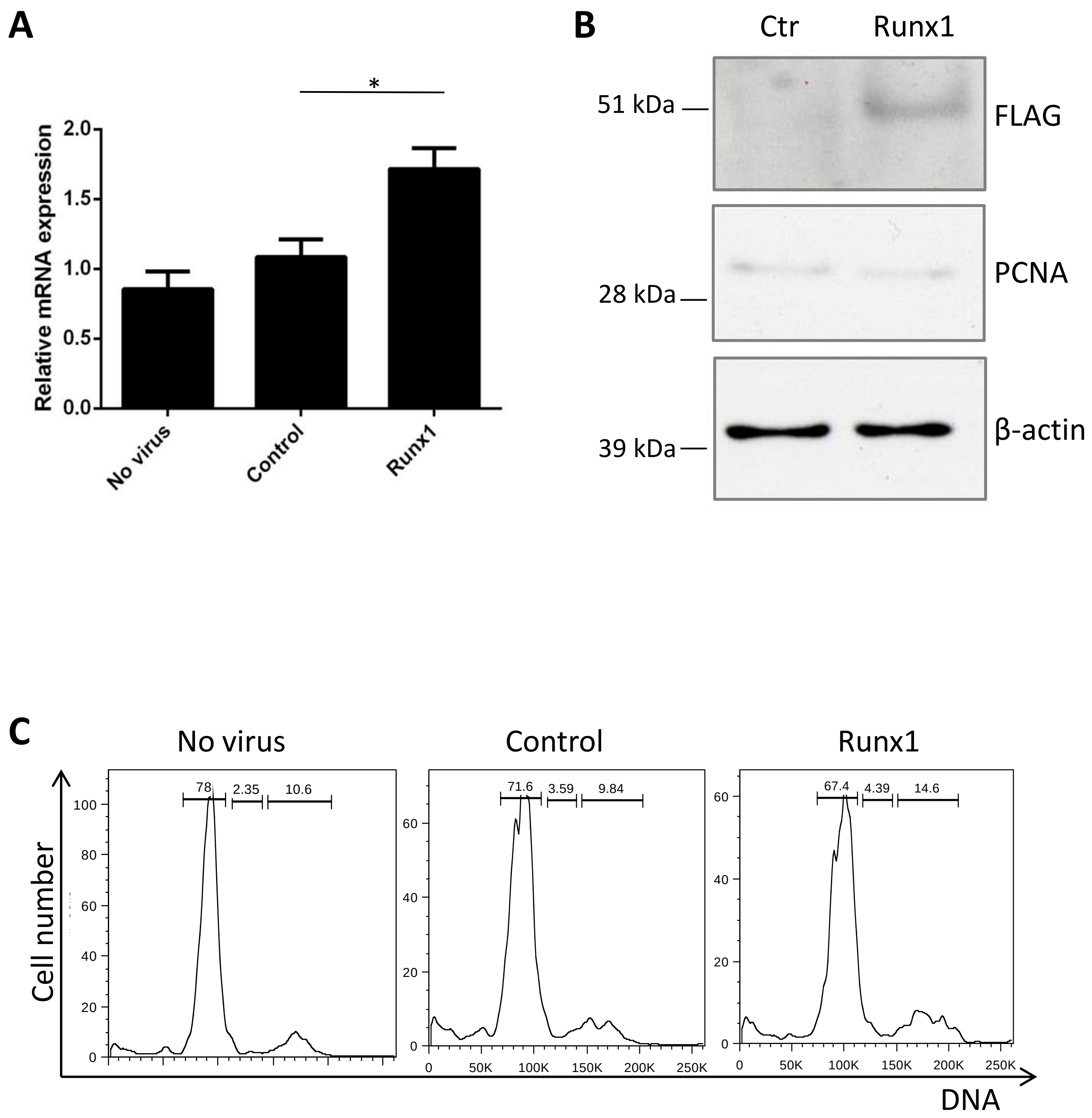

Figure S5. Lentiviral overexpression of *Runx1* in E8.5 ETV2::GFP cells. Related to Figure 6. **A.** Validation of *Runx1* ectopic expression by qRT-PCR. Error bars represent the standard deviation of triplicate values, *p<0.05. **B.** Western blot analysis validates RUNX1b protein expression upon lentiviral transduction. FLAG antibody was used to detect ectopic FLAG-tagged RUNX1b and anti-PCNA antibody was used as a cell proliferation marker. **C.** Cell cycle analysis on fixed cells three day after lentiviral transduction using FxCycle Violet DNA stain.

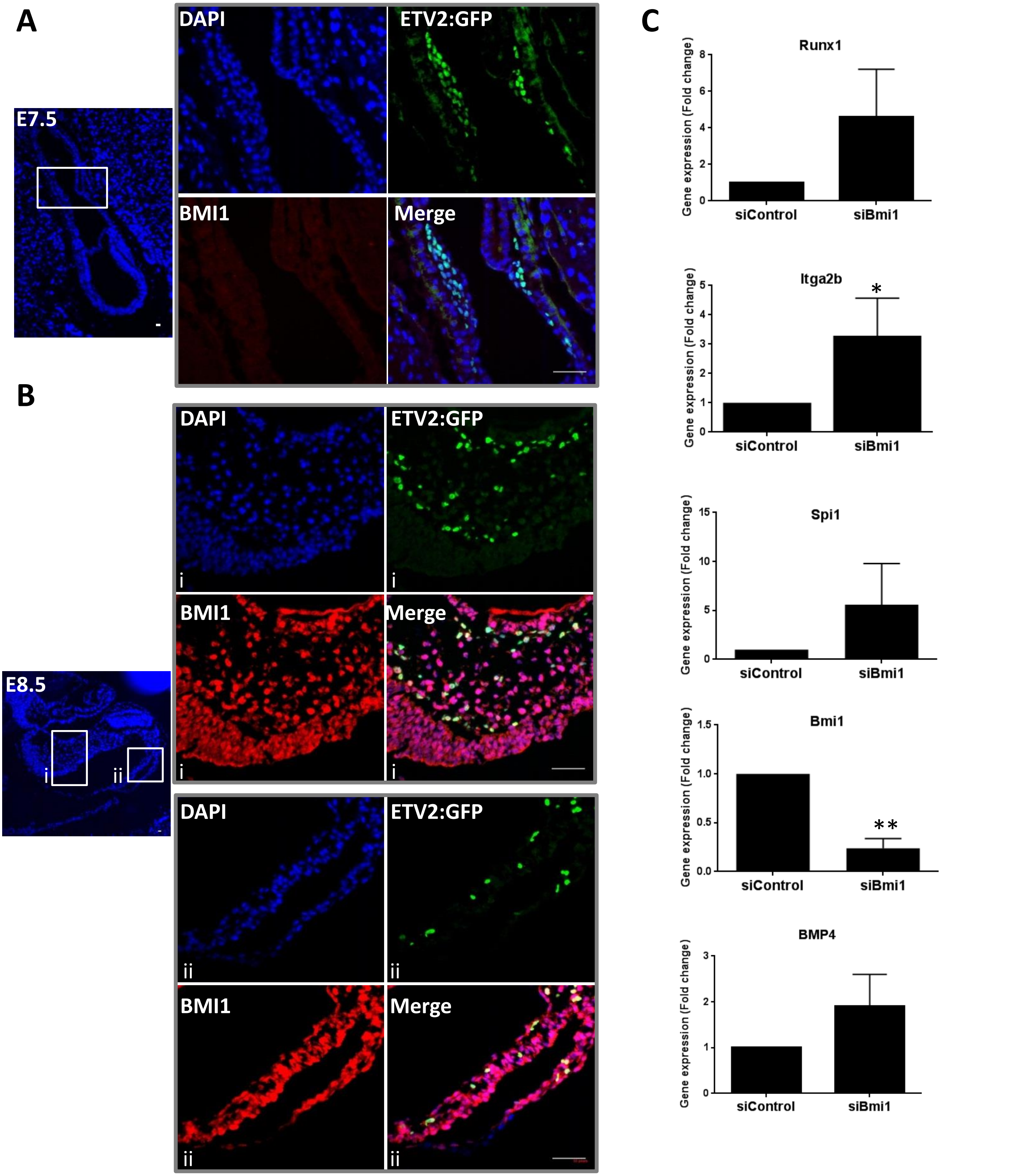

Figure S6. BMI1 expression in ETV2:GFP embryos. Related to figure 7. **A.** Transversal section of *Etv2::gfp* E7.5 and E8.5 embryos (**B**) stained for ETV2::GFP (green) and BMI1 (red). Scale bars=50 µm for whole embryo image and 15 µm for magnified images. Bmi1 staining and imaging were performed under the same conditions for all sections. **C.** Quantitative RT-PCR for E8.5 ETV2::GFP^{+}FLK1^{+}CD41^{-} cells cultured in HE conditions and treated with siControl or siBmi1 for 3 days prior to RNA extraction. Error bars represent 1 SEM for at least three experiments, *p<0.05, **p<0.001.

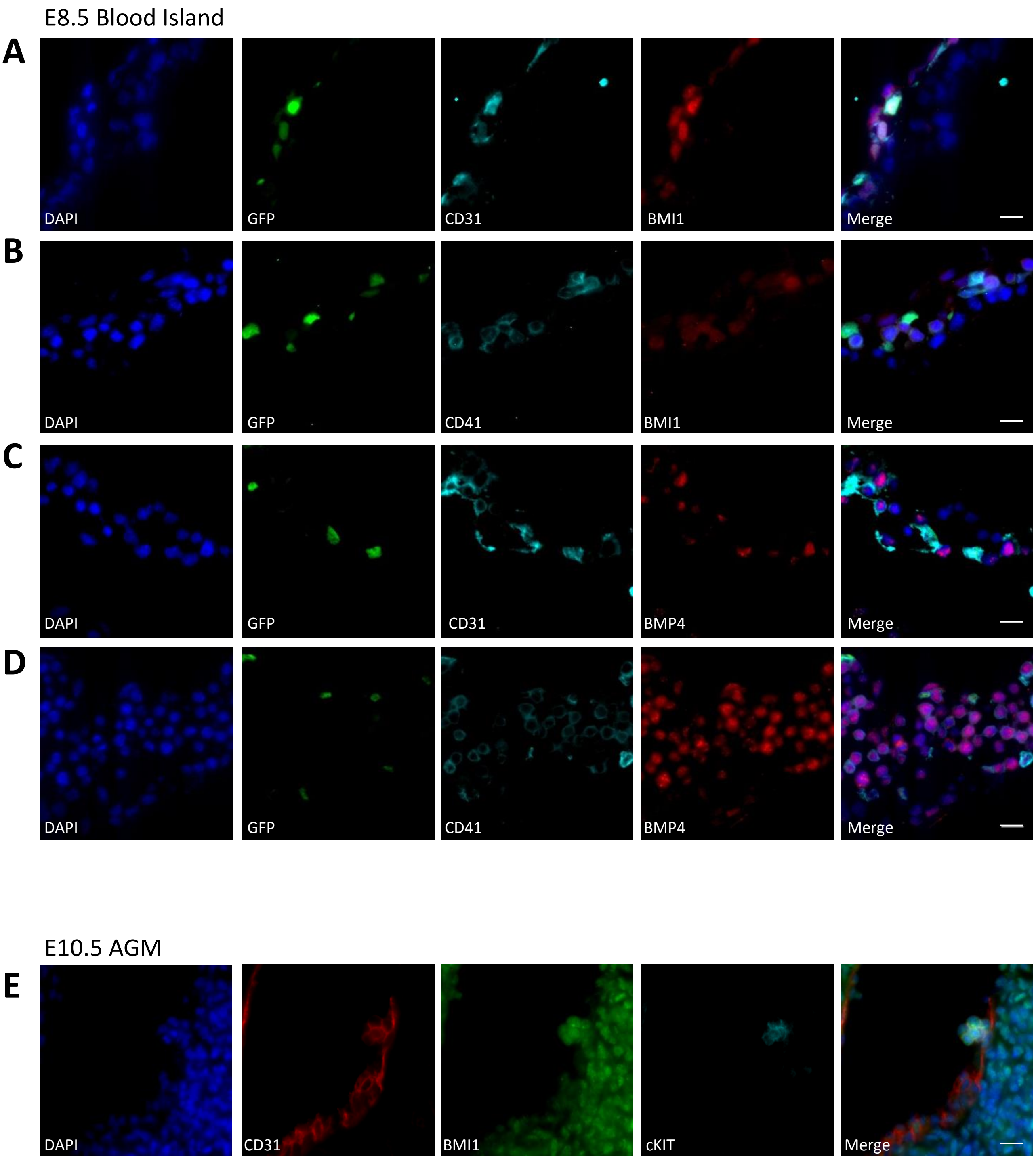

Figure S7. A-D. Transversal section of E8.5 *Etv2::gfp* embryos. Images are focused on blood islands. Sections were stained for DAPI (blue), ETV2::GFP (green), CD41 (cyan), CD31 (cyan), BMI1(red) and BMP4 (red). **E.** Transversal section of E10.5 wild type embryos. Images are focused on the AGM region. Section is stained for DAPI (blue), CD31 (red), BMI1(green) and cKIT(cyan). Scale bar=15µm.

Supplemental movies 1-4. Four examples of live imaging of E7.5 *Etv2::gfp* embryo (Neural plate stage). Relative to Figure S1. 3D reconstruction of z-stacks of transmitted and GFP images. Related to Figure S2.

Supplemental movie 5. 3D reconstruction of z-stacks of E7.5 *Etv2::gfp Runx1b::rfp* embryos stained for CD31 (magenta), ETV2::GFP (green) and RUNX1b::RFP (red). Related to Figure 4.

Supplemental movie 6. 3D reconstruction of z-stacks of E8.5 *Etv2::gfp Runx1b::rfp* embryos stained for ETV2::GFP (green) and RUNX1b::RFP (red). Related to Figure 4.

Supplemental movie 7. Time-lapse imaging of E8.5 $FLK1^{+}GFP^{+}CD41^{-}$ sorted cells on OP9 monolayer cultures transduced with Control and Runx1 lentivirus. Related to Figure 6.

Supplemental Experimental Procedures

Immunofluorescence staining

Immunofluorescence staining was performed on cryo-sections using rat-anti mouse-FLK1 (BD Pharmingen), chicken anti-GFP (Novus Biological), rabbit anti-RFP(MBL), rabbit anti-Smad1/5 and rabbit anti Bmi1 (Cell Signalling) followed by secondary staining with anti-rabbit AlexaFluor555, anti-rabbit AlexaFluor647, anti-rat AlexaFluor647 (Invitrogen) anti-chickenAlexaFluor488 (Jackson Immuno research) and mounted with ProLong Gold antifade reagent with DAPI (Molecular probes). Images were taken with a Low-light ZeisAxiovert 200M system and an AndoriXon DU888+ camera.

Gene expression analysis

Total RNA was extracted using the RNAeasy micro kit and transcribed with Superscript RT kit (Invitrogen) and random hexamer oligos. Real-time PCR was performed using the TaqMan assay (Applied Biosystems) and universal probe library (Roche) and run on an ABI 7900 system (Applied Biosystems).

Microarray analysis

Total RNA was extracted from triplicate biological samples and RNA amplification was performed using the SuperAmp kit preparation kit (Miltenyi). Gene expression analysis was performed using the Affymetrix GeneChip Mouse exon 1.0 ST Array. Gene expression data were processed using RMA (Irizarry et al., 2003) and differential expression was calculated using LIMMA (Smyth, 2004) (Wettenhall and Smyth, 2004). The GEO accession number for the study is GSE64377 (http://www.ncbi.nlm.nih.gov/geo/). Clustering of the Affymetrix samples described in figure 2 and sample GSM721028 from data set GSE29112 was performed after cross-platform normalization. Briefly, a set of genes (n=15786) with probesets represented on the two platforms were identified. For each gene, a gene-level summary was calculated by taking median of the expression of the corresponding probesets. Then cross-platform normalisation method Distance Weighted Discrimination (DWD) as implemented in the R package CONOR (Rudy and Valafar, 2011) (Empirical comparison of cross-platform normalization) was applied to all 6 samples from our Affymetrix experiment and the sample in GSE29112 (GSM721028). Then PCA and hierarchical clustering approaches were used to cluster these samples. The list of endothelial genes plotted in Figure S3 is taken from the Endothelial Cell Biology RT^2 Profiler PCR Array (Qiagen).

Single-cell gene expression analysis

Single-cell gene expression analysis was performed using 96.96 Array (Fluidigm) on the BioMark HD platform. cDNA synthesis and specific gene target amplification was performed using the CellsDirect One-Step qRT-PCR kit (Invitrogen) as previously described (Moignard et al., 2013). The 0.2x assay mix contained a mix of primers from Sigma and probes from the universal probe library (Roche). Raw Ct data were collected from the Fluidigm Data Collection software. Amplification curves were filtered using a threshold of 0.65. Samples not expressing any of the housekeeping genes were excluded from the analysis. Expression values over the cut-off value were set to 26. Hierarchical clustering, PCA plot and violin plots were performed in R either using the Singular analysis toolset 3.0 (Fluidigm) or custom scripts. List of primers used in the specific target amplification is shown below.

Western blot

Proteins were extracted from the cells with radio-immune precipitation assay buffer (RIPA) supplemented with proteinase inhibitors (Roche). Protein concentration was measured with the Bradford assay. Protein lysates were subjected to SDS-PAGE followed by transfer to PVDF membranes. Specific proteins were detected with rabbit anti-FLAG (Sigma), mouse anti-PCNA (BD Biosciences), mouse anti-β actin and rabbit anti-Ubiquitin H2A(Lys119) (Cell Signaling).

Whole mount imaging

For whole mount imaging embryos were processed according to (Yokomizo et al., 2012) using rat anti-mouse CD31 (BD Biosciences), rabbit anti-GFP (MBL), anti-rabbit AlexaFluor488 and anti rat Alexa Fluor647 (Invitrogen). Images were taken using a Leica two photon confocal microscope.

Time-lapse live imaging

E7.5 ETV2::GFP embryos were dissected in DMEM medium containing 5%FCS and 25mM HEPES. Embryos were subsequently cultured in 50% rat serum, 10% FCS, 40% DMEM without phenol red supplemented with 2mM L-Glutamine, 0.1mM 2-ME and 1mM sodium pyruvate. Time-lapse movie acquisition was performed using a Nikon Eclipse Ti confocal microscope equipped with a 20X lense as previously described (Yamanaka et al., 2007). Analysis of cell tracking was performed with IMARIS software (BITTPLANE).

Silencing of Bmi gene by siRNA transfection

Cells sorted from E8.5 embryos were seeded on fibronectin coated plates and transfected with either Silencer select negative control siRNA2 (4390846, Ambion Life technologies) or a combination of 2 siRNAs targeting *Bmi1* (silencer select pre-designed siRNAs, siRNAID:s63005: sense: 5’-GAGCAGAUUGGAUCGAAAtt-3’, antisense: 5’-UUUCCGAUCCAAUCUGCUCtg-3’, siRNAID:s63006: sense: 5’-CGCUAAUGGACAUUGCCUAtt-3’, antisense: 5’-UAGGCAAUGUCCAUUAGCGtg-3’). Transfection was performed using the Lipofectamine RNAiMax reagent (Life technologies) according to the manufacturer’s instructions. 16 hours following transfection the medium was changed and OP9-GFP stroma cells were seeded in the culture to provide support. Two days later the OP9-GFP cells were excluded by FACS sort and the siRNA-treated embryo derived cells were processed for gene expression analysis..

Supplemental References

Rudy, J., and Valafar, F. (2011). Empirical comparison of cross-platform normalization methods for gene expression data. BMC bioinformatics *12*, 467.

Yamanaka, Y., Tamplin, O.J., Beckers, A., Gossler, A., and Rossant, J. (2007). Live imaging and genetic analysis of mouse notochord formation reveals regional morphogenetic mechanisms. Developmental cell *13*, 884-896.

Yokomizo, T., Yamada-Inagawa, T., Yzaguirre, A.D., Chen, M.J., Speck, N.A., and Dzierzak, E. (2012). Whole-mount three-dimensional imaging of internally localized immunostained cells within mouse embryos. Nature protocols *7*, 421-431.

Single Cell gene expression primer list

Primer pairs and Roche universal probes used in single cell-qRT-PCR.

* Primer pairs used for the pre-amplification step but not used in qRT-PCR.

Gene	Sequence FP	Sequence RP	Universal probe
Acta2	atggctctgggctctgtaag	cccattccaaccattactcc	9
Actb	tgacaggatgcagaaggaga	cgctcaggaggagcaatg	106
AI467606	gcgaaatccaagattgaagc	aacagtcaggaacgtctccact	56
Alk1	ctcaagagtcgcaatgtgct	gttgttgccgatatccaggt	76
Bcl2	agtacctgaaccggcatctg	ggggccatatagttccacaaa	75
Bmi1	caaaaccagaccactcctgaa	tcttcttctcttcatctcatttttga	95
Bmp4	gatctttaccggctccagtct	tgggatgttctccagatgttc	63
Cbfb	tatgggttgcctggagtttg	aaggcctgttgtgctaatgc	3
Ccnd3	ggaagatgctggcatactgg	ggtagcgatccaggtagttca	88
Cd34	atgaaccgtcgcagttgg	ccgtgtaataagggtcttcacc	2
Cd79a	gatgccaggggtctagaag	gttcaccgtcagggatgg	91
Cdh5	tcatcaaacccacgaagtcc	ggtctgtggcctcaatgtaga	42
Cdk4	tcagtggtgccagagatgg	ggaaggcagagattcgctta	50
Dll4	aggtgccacttcggttacac	gggagagcaaatggctgata	106
*Ebf1	ccaactcaccctatgccatt	ggggaggcttgtagatgagg	42
Egfl7	catggaggcagcagaggt	tggaacagccagcgtctt	102
Eng	gattccacccagactaaacgtatc	ggttccagtcaggattatctgc	19
Ephb2	gatcctcatgaaagttggacaag	agctctggacgtcttgttgg	72
Erg	agctacgcaaagaattacaactagg	ttggactgaggggtgaggt	64
Ets1	ctgacctcaacaaggacaagc	agaaactgccacagctggat	18
Etv2	cattgactcgctactccaaaact	tggaactcgcggctattg	79
F2r	gtcttcccgcgtccctat	gggttcaccgtagcatctgt	20
Fli1	agaccatggcaagaacact	gccccaggatctgataagg	20
Foxf1	agcatctccacgcactcc	tgtgagtgataccgagggatg	46
Gapdh	agcttgtcatcaacgggaag	tttgatgttagtggggtctcg	9
Gata1	ccctgaactcgtcataccact	gaacactggggttgaacctg	83
Gata2	gcttcacccctaagcagaga	tggcaccacagttgacaca	15
Gata3	ttatcaagcccaagcgaag	tggtggtggtctgacagttc	108
Gfi1	atgtgcggcaagaccttc	acagtcaaagctgcgttcct	1
Gfi1b	agcacagagtctcccttgga	atgaggggtggagaacacc	80
GFP	gaagcgcgatcacatggt	ccatgccgagagtgatcc	67
Gpr56	attgctgcctacctctgctc	ggacagcaggttcatgtgg	69
Hbb	tggatcctgagaacttcaagc	attggccactccaatcacc	29
Hes1	acaccggacaaaccaaagac	cgcctcttctccatgatagg	99
Hhex	actacacgcacgccctactc	cctttcctttgtgcagagg	69
Hoxb3	ctggggctcgatgtgaata	ctgttccaagcggctgac	96
Hoxb4	ctggatgcgcaaagttcac	gtgaaactccttctccaactcc	62
Hprt1	tcctcctcagaccgctttt	cctggttcatcatcgctaatc	95
Icam1	cccacgctacctctgctc	gatggatacctgagcatcacc	81
Icam2	ttgctggagcctgtctcttc	ctcaaaggccttctcaccag	26
Ier2	aagtagcgtcccccgaag	gcacttcctcttgcgtatcc	75
Ikzf1	gacatgtcccaagtttcagga	gcctcgatcactcttggagtt	99
Ikzf2	cacaactatctccagaatgtcagc	caatcttccataggcggtaca	75
Itga2b	tgctgctgaccctgctagt	gtcgattccgcttgaagaag	97
Itga3	tcaacatggagaacaagacca	ccaaccacagctcaatctca	25
Itgam	caatagccagcctcagtgc	gagcccaggggagaagtg	76
Itgav	ggagggcaagttctcaaatg	ctttggcataatctcgattgc	42
Itgb3	tgaccggaaggaatttgcta	acagcgggttgtttgctg	21
Jag1	tggccgaggtcctacactt	gccttttcaattatgctatcagg	22
Jag2	cgtcattccctttcagttcg	cctcatctggagtggtgtca	95
Kdm1a	ctcctggcccctcaattc	tgtgtgttctccagcaaagaa	76
Kdr	aaagcgggacgaggagag	caggttgcacagtaatttcagg	12
Kit	acaaattcaccctcaaagtgc	ggtgacttgtttcaggcaca	46
Lama3	cacacctgggacgtggat	gaagcagcaccaggtagtcc	12

Single Cell gene expression primer list (continued)

Lgr5	agcagactacgcctttggaa	tcccagggagtggattctatta	19
Lmo2	cgaaaggaagagcctggac	cggtcccctatgttctgct	83
Lyl1	gcaggacccttcagcatct	ccaccttctggggttggt	41
Mcm6	ctccacctcaggaaaatgga	ttgacaagctcgcttctcttt	101
Mecom	cgcttgaagctttgaaagaaaa	tgccatgttctcaattgctg	80
Mesp1	acccatcgttcctgtacgc	agcatgtcgctgctgaaga	89
Mki67	gctgtcctcaagacaatcatca	ggcgttatcccaggagact	80
Mpo	gatggaatggggagaagctc	gcaggtagtcccggtatgtg	79
Myb	ctgtcaaaagcctacccttctc	tggagggtaaggtaggtgcat	11
Myl9	gataaggaggacctgcacga	ggaacatggtgaagttgatgg	60
Mylk	atagccttggccaggtgtc	gctgtctgtgccaagttcg	40
Nanog	caggtttcagaagcagaagtacc	ggttttgaaaccaggtcttaacc	67
Nfe2	acactacccgcagcctcat	cacaaaggattcctgcaacc	91
*Nkx2-5	gacgtagcctggtgtctcg	gtgtggaatccgtcgaaagt	53
Notch1	ctggaccccatggacatc	aggatgactgcacacattgc	80
Notch2	tgcctgtttgacaactttgagt	gtggtctgcacagtatttgtcat	6
Notch4	ggacctgcttgcaaccttc	cctcacagagcctcccttc	34
PCNA	ctagccatgggcgtgaac	gaatactagtgctaaggtgtctgcat	41
Pecam1	gctggtgctctatgcaagc	atggatgctgttgatggtga	64
*PF4	catctcctctgggatccatct	ccattcttcagggtggctat	9
Plau	gccttggtggtgaaaaactc	cacgcatacacctccgttc	5
Pou5f1	ctcccatgcattcaaactga	ttctcttgtctacctcccttgc	100
Rrocr	agcgcaaggagaacgtgt	gggttcagagccctcctc	26
PTPRC	tcagaaaatgcaacagtgacaa	ccaactgacatctttcaggtatga	100
Rpl19	ctcgttgccggaaaaaca	tcatccaggtcaccttctca	103
Runx1 (exon8)	cggttcctaccagttctcca	tctccaccacgtcgctct	75
Runx1 (Runt domain)	ctccgtgctacccactcact	atgacggtgaccagagtgc	77
Runx1_distal	gaagtgtaagcccagcacagt	ggcgggggattctataattt	40
Runx1_proximal	aagatccgagcccctgtc	tcacaacaagccgattgagt	17
Runx2	gcccaggcgtatttcagat	cacctgcctggctcttctta	34
Runx3	ttcaacgaccttcgattcgt	ttggtgaacacggtgattgt	103
Snai1	cttgtgtctgcacgacctgt	caggagaatggcttctcacc	71
Sox17	cacaacgcagagctaagcaa	cgcttctctgccaaggtc	97
Sox18	cgcagtactgagcaagatgc	cggtacttgtagttgggatggt	12
Sox2	ggcagagaagagagtgtttgc	tcttctttctcccagcccta	34
Sox7	cagcaagatgctgggaaag	tgcatcatccacatagggtct	97
Spi1	ggagaagctgatggcttgg	caggcgaatctttttcttgc	94
T	cagcccacctactggctcta	gagcctggggtgatggta	100
Tal1	gctcgcctcactaggcagt	cttcacccggttgttgttg	60
Tcf7L1	ctgagcagcccgtacctct	aggggccatttcatctgtag	22
Tek	tgcccagatattggtgtcct	cagtgtggtgttcacgtatgtct	89
Tert	tgaaagtagaggattgccactg	cctcagacggtgctctgc	66
Ubc	gtctgctgtgtgaggactgc	cctccagggtgatggtctta	77
Unc5c	aggcagtgcagggacaat	aatgtaacaggggttgtgaa	52
*Vwf	gagaatgcagacccaccttt	ggggacactcttttgcactc	98
Was	caccaccagcaccaatca	tcctagagatagtggaccacctg	91
