# Supplementary figures and images for: The Hemogenic Competence of Endothelial Progenitors Is Restricted by Runx1 Silencing during Embryonic Development
Source: Cell Rep. 2016 May 26;15(10):2185–99. doi: 10.1016/j.celrep.2016.05.001 (PMC4906370; doi:10.1016/j.celrep.2016.05.001)

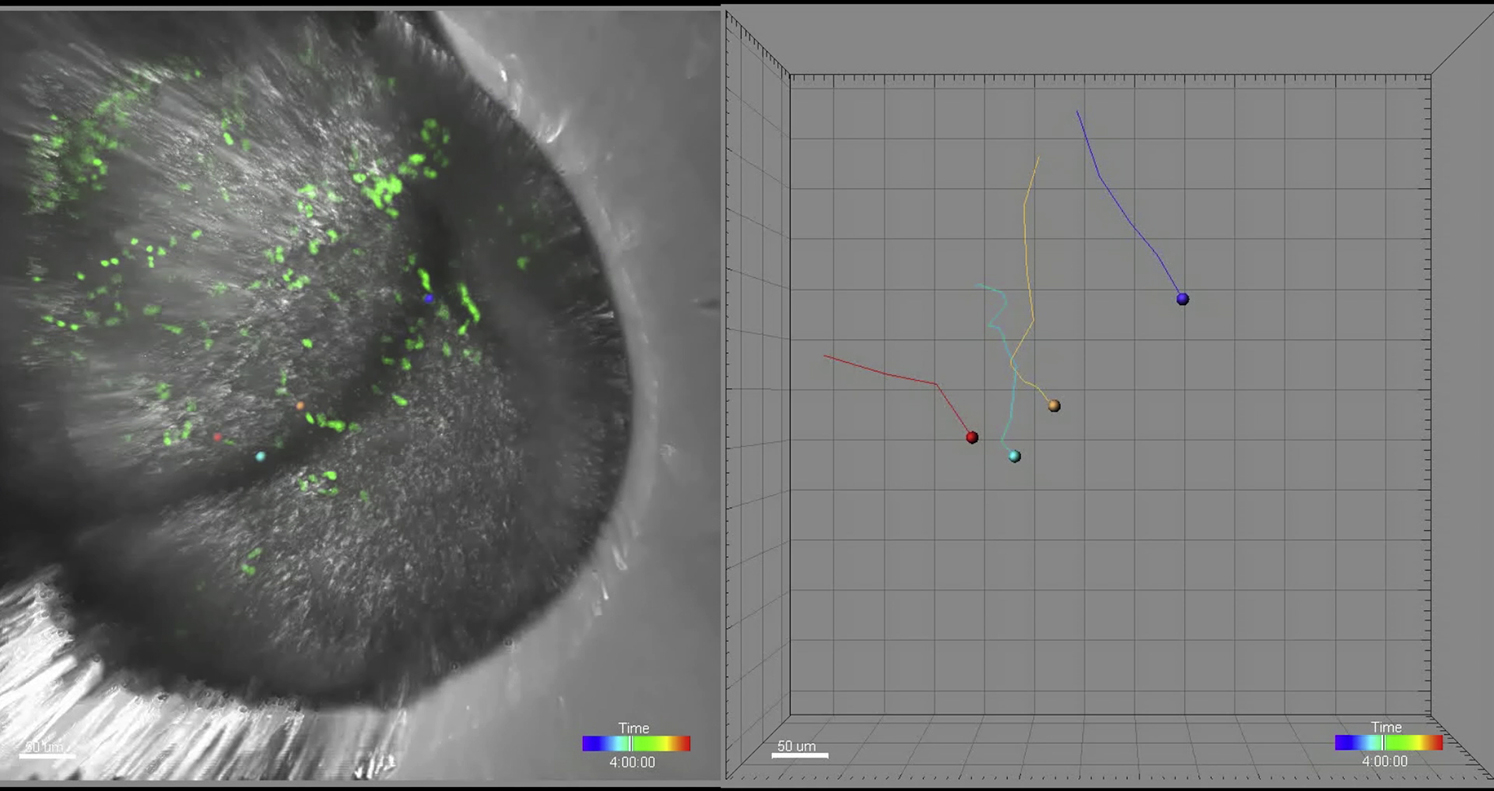

Supplement: Movie S1. Example 1, Live imaging of E7.5 Etv2::gfp embryo, Related to Figure S2 [file mmc2.jpg]

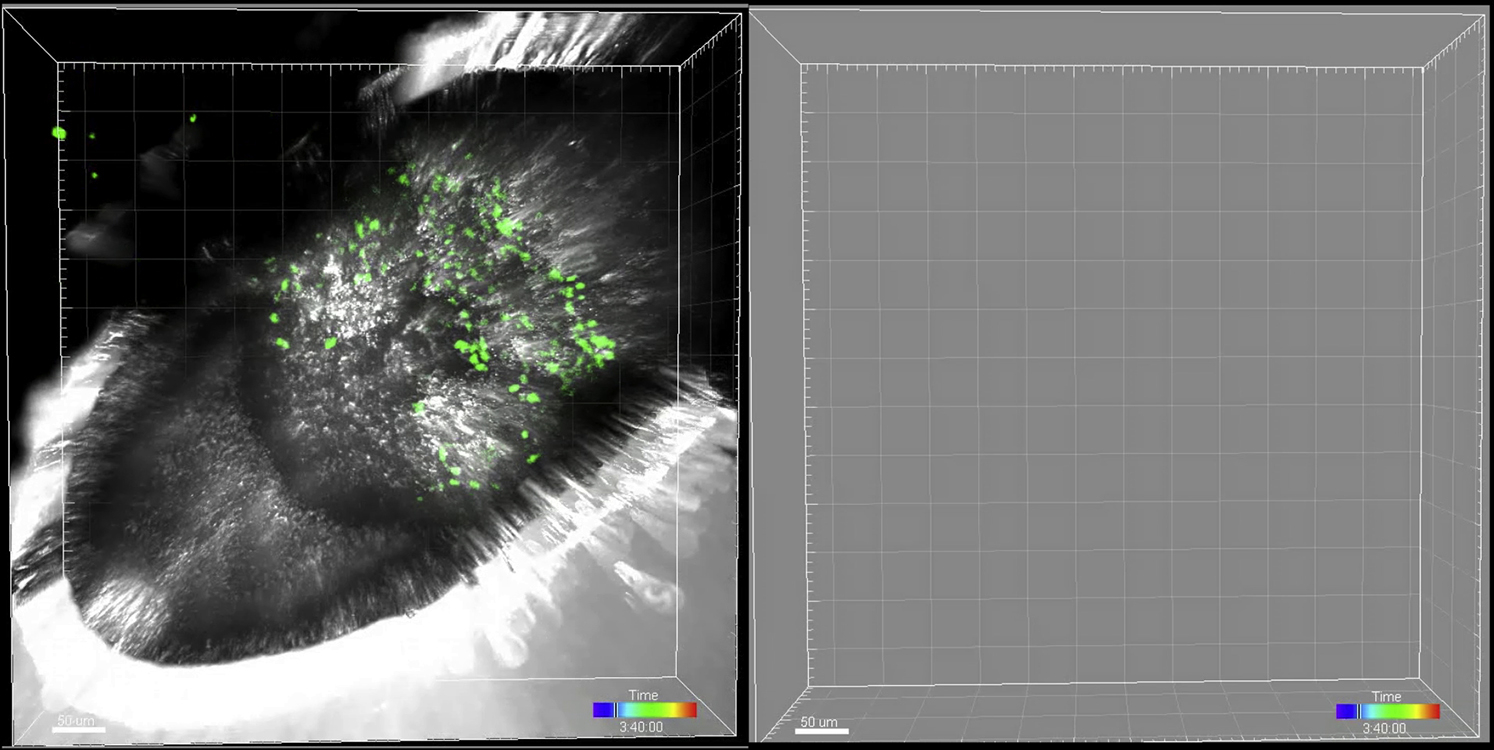

Supplement: Movie S2. Example 2, Live imaging of E7.5 Etv2::gfp embryo, Related to Figure S2 [file mmc3.jpg]

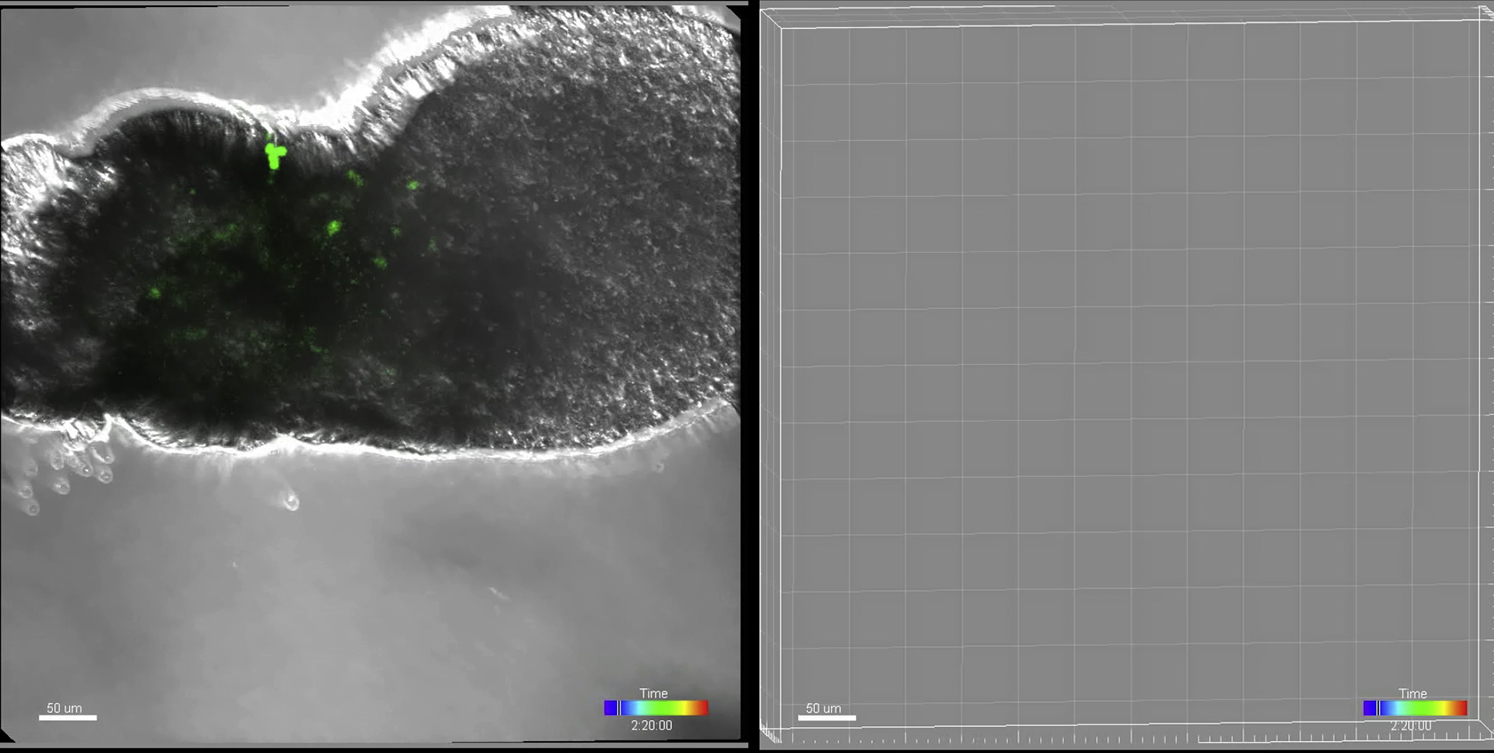

Supplement: Movie S3. Example 3, Live imaging of E7.5 Etv2::gfp embryo, Related to Figure S2 [file mmc4.jpg]

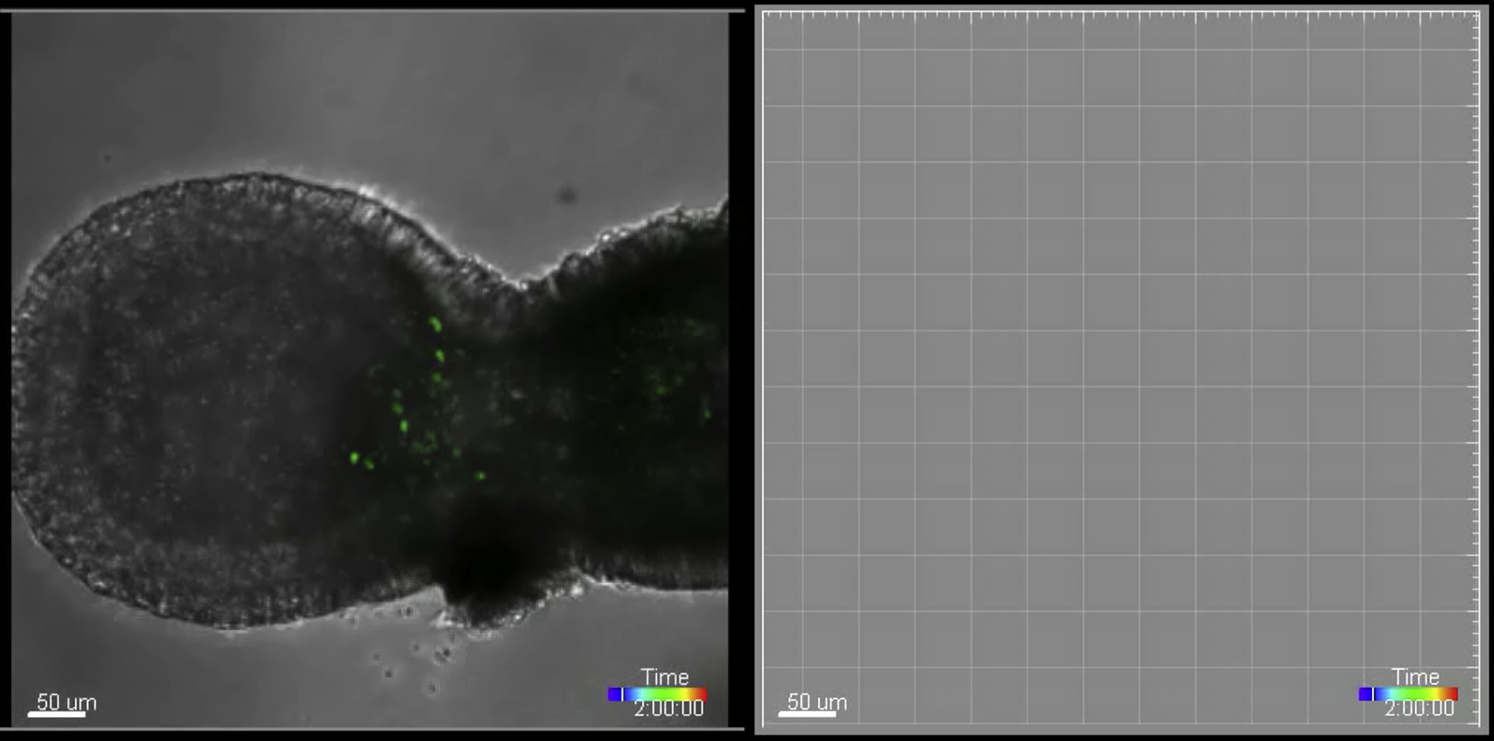

Supplement: Movie S4. Example 4, Live imaging of E7.5 Etv2::gfp embryo, Related to Figure S2 [file mmc5.jpg]

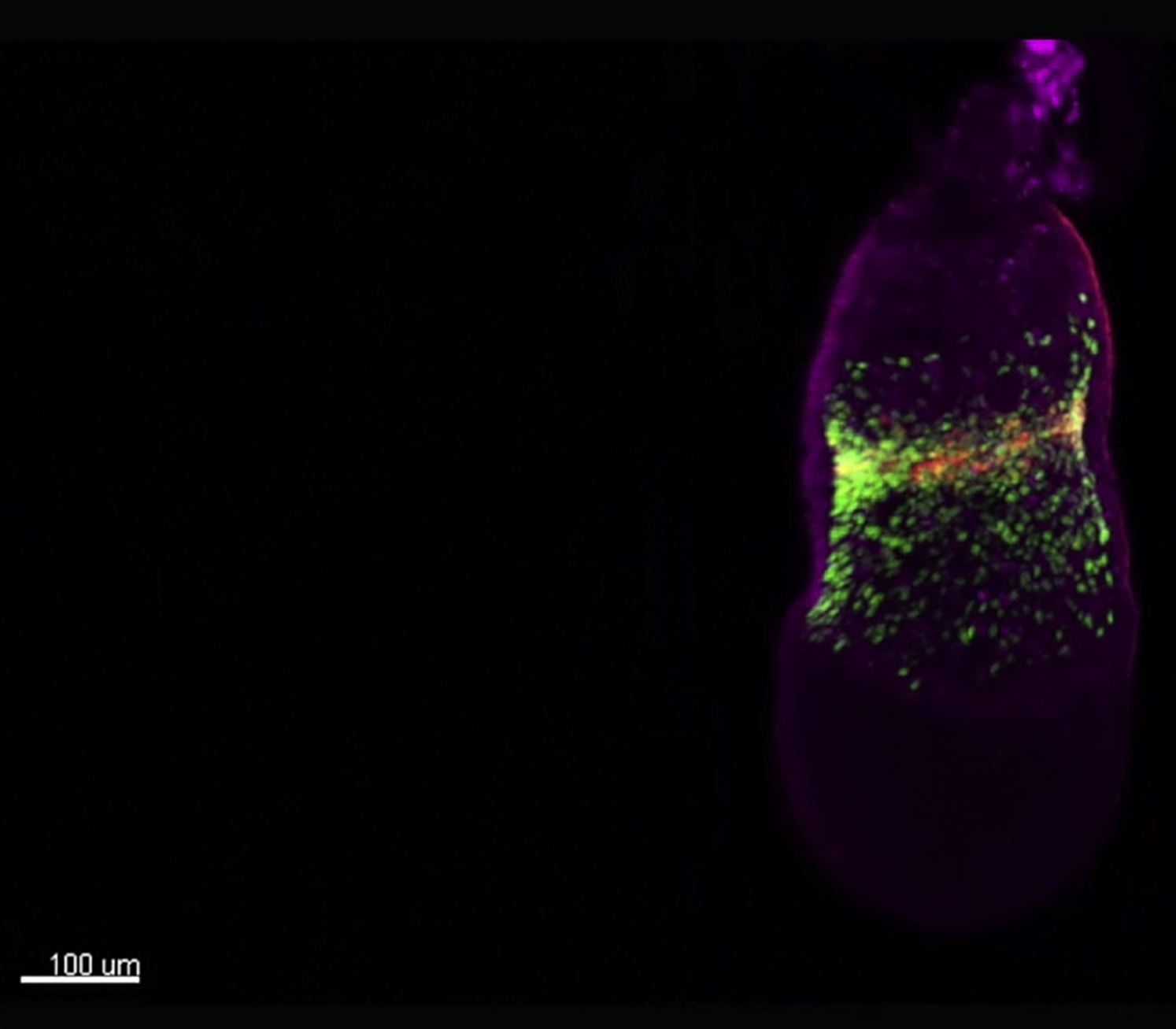

Supplement: Movie S5. 3D Reconstruction of z Stacks of E7.5 Etv2::gfp Runx1b::rfp Embryos Stained for CD31, in Magenta; ETV2::GFP, in Green; and RUNX1b::RFP, in Red, Related to Figure 4 [file mmc6.jpg]

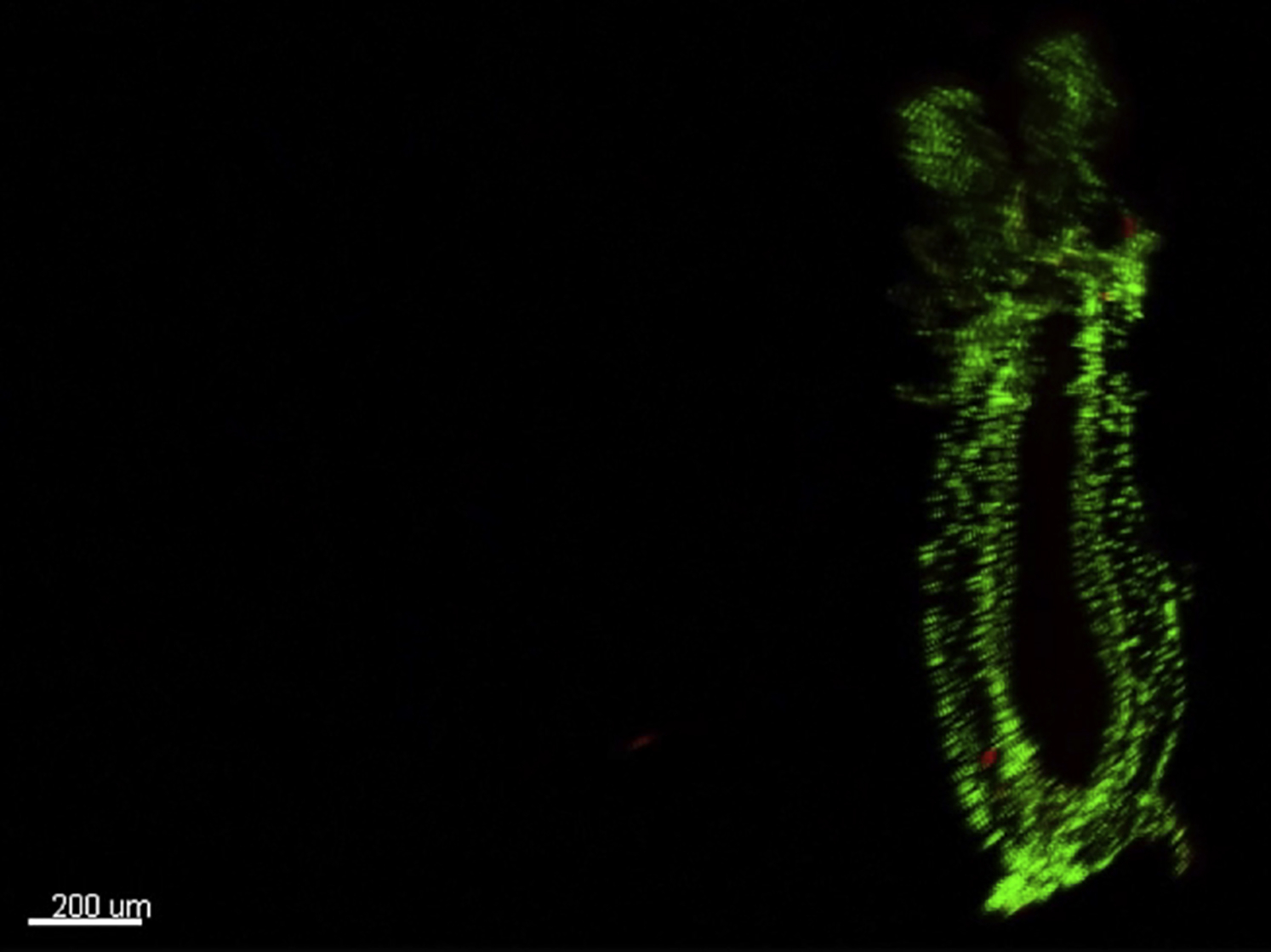

Supplement: Movie S6. 3D Reconstruction of z Stacks of E8.5 Etv2::gfp Runx1b::rfp Embryos Stained for ETV2::GFP, in Green, and RUNX1b::RFP, in Red, Related to Figure 4 [file mmc7.jpg]

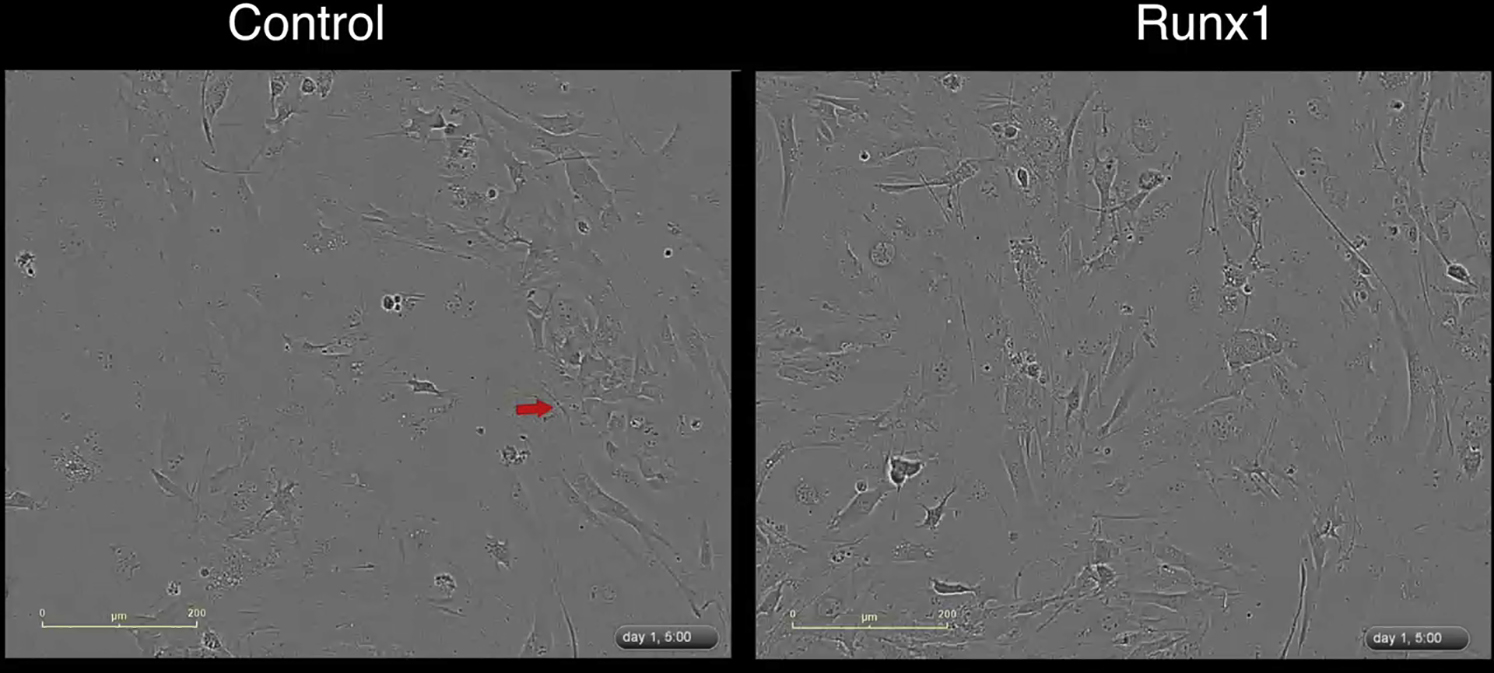

Supplement: Movie S7. Time-Lapse Imaging of E8.5 FLK1+GFP+CD41− Sorted Cells on OP9 Monolayer Cultures Transduced with Control and Runx1 Lentivirus, Related to Figure 6 [file mmc8.jpg]
